# Single-cell RNA sequencing reveals induction of distinct trained-immunity programs in human monocytes

**DOI:** 10.1172/JCI147719

**Published:** 2022-04-01

**Authors:** Bowen Zhang, Simone J.C.F.M Moorlag, Jorge Dominguez-Andres, Özlem Bulut, Gizem Kilic, Zhaoli Liu, Reinout van Crevel, Cheng-Jian Xu, Leo A.B. Joosten, Mihai G. Netea, Yang Li

**Affiliations:** 1 Department of Computational Biology of Individualised Medicine, Centre for Individualised Infection Medicine (CiiM) and; 2 TWINCORE, Centre for Experimental and Clinical Infection Research, a joint venture between the Hannover Medical School and the Helmholtz Centre for Infection Research, Hannover, Germany.; 3 Department of Internal Medicine and Radboud Center for Infectious Diseases, Radboud University Medical Center, Nijmegen, Netherlands.; 4 Department of Medical Genetics, Iuliu Haţieganu University of Medicine and Pharmacy, Cluj-Napoca, Romania.; 5 Department for Genomics & Immunoregulation, Life and Medical Sciences Institute (LIMES), University of Bonn, Bonn, Germany.

**Keywords:** Immunology, Infectious disease, Innate immunity, Macrophages, Monocytes

## Abstract

Trained immunity refers to the long-lasting memory traits of innate immunity. Recent studies have shown that trained immunity is orchestrated by sustained changes in epigenetic marks and metabolic pathways, leading to an altered transcriptional response to a second challenge. However, the potential heterogeneity of trained-immunity induction in innate immune cells has not been explored. In this study, we demonstrate cellular transcriptional programs in response to 4 different inducers of trained immunity in monocyte populations at single-cell resolution. Specifically, we identified 3 monocyte subpopulations upon the induction of trained immunity, and replicated these findings in an in vivo study. In addition, we found gene signatures consistent with these functional programs in patients with ulcerative colitis, sepsis, and COVID-19, suggesting the impact of trained-immunity programs in immune-mediated diseases.

## Introduction

Host immune responses are classically divided into innate immune responses and adaptive immune responses. In recent years, emerging evidence has shown that innate immunity can display long-term adaptive characteristics after challenge with certain endogenous or exogenous stimuli, and display a de facto nonspecific immunological memory, termed “trained immunity” (1). Although the induction of long-term functional reprogramming in prototypical innate immune cells (such as monocytes, macrophages, and NK cells) results in qualitatively different transcriptional responses upon secondary stimulation, a protective role of trained immunity (TI) has been suggested to contribute to the defense against a broad array of infections, including SARS-CoV-2 (2). Epigenetic and metabolic reprogramming of innate immune cells, including histone methylation and acetylation, were reported to mediate this process (3–5).

One well-studied aspect of the TI phenotype is an enhanced production capacity by monocytes of the proinflammatory cytokines IL-1β, TNF-α, and IL-6 in response to a secondary stimulation (6, 7). A recent study has also reported CXCL9, CXCL10, and potentially CXCL11 as markers of TI (8). Several stimuli, such as β-glucan (BG, part of the cell wall of *Candida* *albicans*), bacillus Calmette-Guérin (BCG) vaccine, uric acid (UA) (9), muramyl dipeptide (MDP, part of the cell wall of mycobacteria) (7), and oxidized low-density lipoprotein (oxLDL) have been shown in various studies to efficiently induce TI in monocytes/macrophages (10). In the meantime, extensive studies have demonstrated induction of TI after BCG vaccination in healthy individuals (11, 12), and it has been hypothesized that TI mediates protective heterologous effects after live attenuated vaccines (13).

The enhanced immune responses of TI were also reported at the level of the transcriptome. Both Cheng et al. (4) and Mitroulis et al. (14) described genes that are involved in several innate immune functions and pathways of cell metabolism, and which were found upregulated in BG-treated mice. Several metabolic pathways such as the Akt/mTOR/HIF-1α signaling pathway and NOD2-receptor pathway have been shown to be switched on by TI (3, 4). The induction of TI is also associated with functional differences, as we have shown in previous studies (15, 16).

The consistency and variations of training efficiency highlight the complexity of systemic immune responses in TI. However, the potential heterogeneity in the transcriptional responses of innate immune cells involved in TI in a certain individual has not been studied to the best of our knowledge. For instance, do all monocytes show similar gene expression profiles upon the induction of TI or is there heterogeneity across cells? Does the induction of TI by different stimuli result in distinct transcriptional programs?

To answer these questions, we performed single-cell RNA sequencing (scRNA-seq) in monocytes that were trained in vitro by 4 different stimuli (10). Characterization of gene expression profiles revealed a high heterogeneity of TI hallmark cytokines and chemokine markers across monocyte subpopulations. Specifically, we identified 3 shared types of transcriptional programs in monocytes trained by any of the 4 stimuli and further investigated their functional enrichments in molecular pathways after restimulation with the microbial stimulus lipopolysaccharide (LPS). Finally, we validated the signature expression profiles of these monocyte subpopulations both in sepsis and COVID-19 patients, as well as in individuals that were trained in vivo by a BCG vaccination (Supplemental Figure 1; supplemental material available online with this article; https://doi.org/10.1172/JCI147719DS1). Altogether, our study presents a comprehensive view of cellular transcriptional programs of TI at single-cell resolution, which outlines the heterogeneity in gene expression in trained cells. These discrete, trained populations of monocytes may provide novel insight into the molecular mechanism of TI and its role in immune-mediated diseases.

## Results

### scRNA-seq profiling of trained monocytes and macrophages.

**Figure 1:**
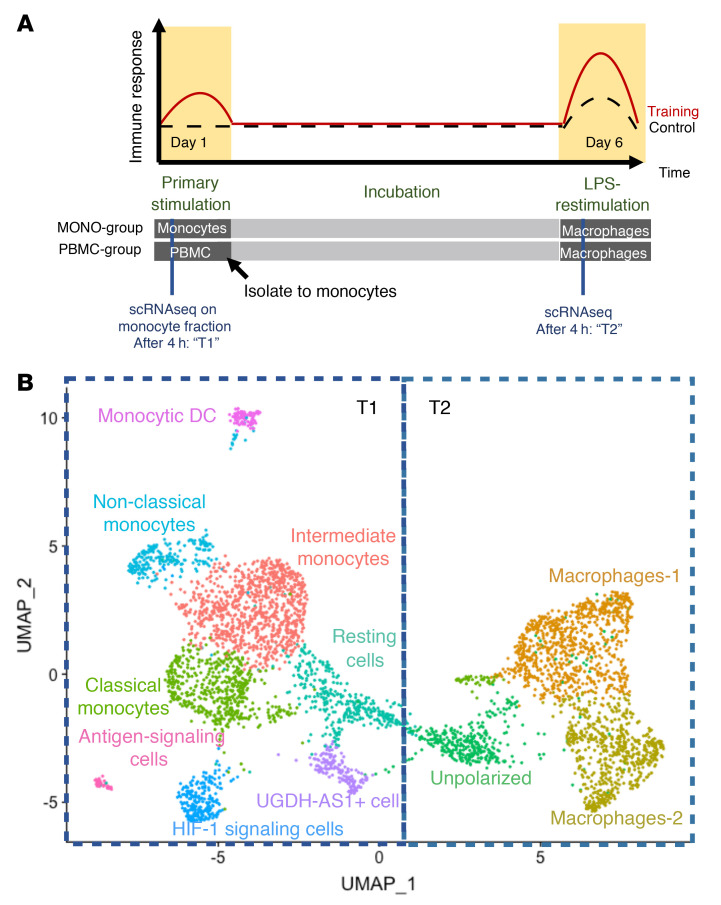
Single-cell expression atlas and cluster annotations in monocytes and macrophages of training and control samples. (**A**) Study design. Monocytes (M-MONO) and PBMCs (M-PBMC) were isolated and incubated in vitro with culture medium (RPMI, negative control), β-glucan (BG), uric acid (UA), oxidized low-density lipoprotein (oxLDL), and muramyl dipeptide (MDP) for 24 hours. After a 4-hour stimulation, cells were isolated for scRNA-seq (T1). On day 6, cells were restimulated with LPS for 4 hours and then isolated for scRNA-seq (T2). M-MONO, monocytes trained in the absence of lymphocytes; M-PBMC, monocytes trained in the presence of lymphocytes. (**B**) UMAP of cells from RPMI control and training conditions. Cells are colored by unsupervised clusters, with corresponding cell type annotated based on known cell-type-specific marker genes.

To study the transcriptomic profiles of trained monocytes, we used a previously reported in vitro model of TI (10). Briefly, blood samples (see Supplemental Table 1 for sample information) were drawn from 3 healthy human individuals and isolated monocytes (M-MONOs) were incubated in vitro for 24 hours with culture medium (negative control) or 4 different well-known inducers of TI: BG (1 mg/mL; ref. 16), UA (10 mg/mL; ref. 17), oxLDL (10 mg/mL; ref. 18), and MDP (1 mg/mL; ref. 7). We used BG and MDP to mimic TI induced by fungal cells and mycobacteria, respectively, while UA and oxLDL were used as endogenous inducers of TI in inflammatory disorders. Additionally, in order to explore a potential effect of the presence of lymphocytes on gene expression in monocytes, we stimulated PBMCs in the first 24 hours of training stimulation (M-PBMCs). After 24 hours, the stimuli were removed and isolated monocytes were rested and incubated for 5 days in culture medium. On day 6, cells from all conditions were restimulated with LPS for 4 hours, after which RNA was isolated to assess the transcriptome (Figure 1A). Using the single-cell SORT-seq technique (19), we profiled the transcriptomic profile of 4,362 monocytes/macrophages at both 4 hours after the first stimulation (T1) and upon restimulation with LPS on day 6 (T2). As shown in Figure 1B, the uniform manifold approximation and projection (UMAP) plot revealed a distinct separation between cells from T1 and T2. All clusters of cells were uniformly distributed among donors, suggesting little donor-related batch effects (Supplemental Figure 2). Unsupervised clustering analysis identified in total 11 subpopulations.

**Figure 2:**
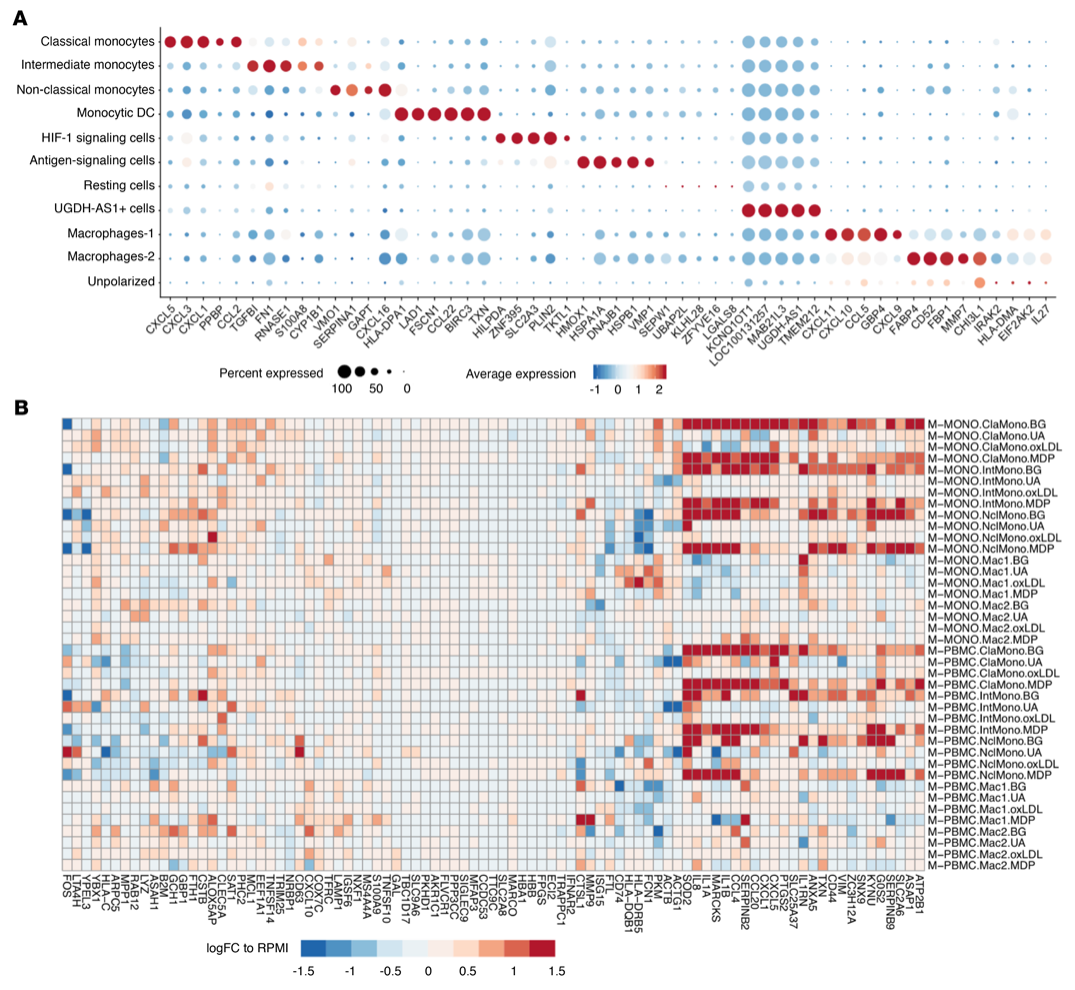
Differentially expressed genes identified in monocytes and macrophages of training and control samples. (**A**) Dot heatmap shows the top 5 differentially expressed genes (DEGs) in each cluster. DEGs were obtained by comparing expression level in cells of one cluster to that in the rest of cells. (**B**) Average log(fold change) relative to RPMI controls of each group across 3 monocytes and 2 macrophages. ClaMono, classical monocytes; IntMono, intermediate monocytes; NclMono, nonclassical monocytes; Mac1, Macrophages-1; Mac2, Macrophages-2.

In order to systematically identify cell-subpopulation-specific marker genes, we performed differential expression (DE) analysis by comparing the expression level in cells of one cluster to the levels in the rest of the cells (Figure 2A and Supplemental Table 2A). Based on the top differentially expressed genes (DEGs) and known cell-type-specific marker genes (Supplemental Figure 3A), we were able to annotate 11 subpopulations: 3 monocyte subsets as classical, intermediate, and nonclassical monocytes and a small cluster as monocyte-derived dendritic cells for the cells of T1; 2 macrophage subsets (Macrophages-1 and -2) for the cells of T2, and 2 clusters of cells labeled as resting cells and unpolarized macrophages, as they were located in the transition phase between cells of T1 and T2 in the UMAP and showed overall low gene expression; 3 small subpopulations mainly detected at T1 were labeled as “HIF-1 signaling cells,” “antigen-signaling cells,” and “UGDH-AS1 cells” based on the Kyoto Encyclopedia of Genes and Genomes (KEGG) enrichment terms of their expressed genes (Supplemental Figure 3B). The majority of identified cell clusters were consistently present across stimulation environments (M-PBMC/M-MONO) and conditions (RPMI, BG, UA, oxLDL, or MDP; Supplemental Figure 2).

### Impact of training stimuli on cell frequency and marker gene expression.

To gain an overview of both the shared and unique characteristics of monocytes trained by 4 different stimuli and RPMI control, we compared the cell frequency and marker gene expression of the identified cell subsets among the different conditions (Supplemental Figure 3C). Cell frequencies of different subsets from each condition were calculated at both T1 and T2. At T1, the most abundant cell types were classical, intermediate, and nonclassical monocytes, whereas at T2, Macrophages-1 and -2 were found to be the major cell types. These observations are in line with the literature (20, 21). Of note, the frequency of those major cell types showed no significant difference in all 4 training conditions and control condition, suggesting a similar impact on the relative abundance of monocyte subpopulations.

Next, we investigated the DEGs by comparing them to RPMI controls of each cell type across the 4 training conditions (Figure 2B and Supplemental Table 2B). The majority of these marker genes showed similar regulation directions across training conditions. Specifically, in monocytes at the T1 time point, *PTGS2*, *ATP2B1*, and *MHC* class II gene expression was more strongly induced by all stimuli compared with RPMI controls, while a significantly higher expression of *CCL4* (BG, *P_adj_* = 1.41 × 10^–21^; MDP, *P_adj_* = 9.45 × 10^–21^), *IL1B* (BG, *P_adj_* = 2.12 × 10^–22^; MDP, *P_adj_* = 1.86 × 10^–26^), *IL1A* (BG, *P_adj_* = 1.21 × 10^–13^; MDP, *P_adj_* = 6.29 × 10^–6^) was seen in BG- and MDP-stimulated cells, and *IL1RN* (*P_adj_* = 1.65 × 10^–29^) in BG. The top DEGs in each training stimulus compared with nontrained control followed similar patterns in the M-MONO and M-PBMC groups (Figure 2B). Additionally, most significant DEGs were found upregulated in BG- and MDP-stimulated conditions (Supplemental Figure 4A and Supplemental Table 2B). They showed Gene Ontology (GO) pathway enrichment of cellular response to IL-1 and biotic stimuli, and KEGG pathway enrichment in IL-17, TNF, NF-κB, and Toll-like receptor signaling pathways (Supplemental Figure 4B), which are very important components of the innate-immune and inflammatory responses during host defense. These results suggest that higher inflammatory responses were induced by BG and MDP than UA and oxLDL.

At T2, classical or nonclassical monocyte markers *CD14* and *FCGR3A* (*CD16*) were minimally expressed across all conditions, while macrophage markers (e.g., *CD83*, *CD36*, *TNF*, *IL1B*, *STAT1*, and *IFI6*; Supplemental Figure 3A), including tissue residence markers *PTPRC* (CD45, *P_adj_* = 3.69 × 10^–36^ in unpolarized), *SIGLEC1* (CD169, *P_adj_* = 5.95 × 10^–244^ in Macrophages-1), and *KLF4* (*P_adj_* = 8.35 × 10^–82^ in Macrophages-1), were highly expressed at this time point in all conditions. These changes suggest that, regardless of stimuli conditions, most monocytes differentiated toward macrophages during the incubation steps. When comparing the various TI conditions with the RPMI control, only a small number of genes were found upregulated (Supplemental Figure 4A and Supplemental Table 2B). These data suggest that the TI transcriptional program induced by the various stimuli is independent of the process of macrophage differentiation, which occurs independently in all conditions.

### Heterogeneous expression of TI signature genes in macrophages.

We hypothesized that, at the single-cell transcriptional level, TI characteristics may be heterogeneous. Previous studies have shown that the induction of TI leads to an enhanced production capacity of key proinflammatory cytokines (IL-1β, IL-6, and TNF-α) and chemokines (*CXCL9-11*) in monocytes (8). Therefore, we wanted to investigate the transcriptional programs that underlie these important functional changes, and we therefore focused on these TI signature genes (*IL1B*, *IL6*, *TNF*, and *CXCL9-11*) and investigated their expression distribution across all cells. Interestingly, *TNF* and *CXCL9-11* were only expressed at T2, whereas *IL1B* was expressed at both T1 and T2. Moreover, we observed a large variation in the expression levels of these signature genes across cells in both the M-MONO and M-PBMC groups (Supplemental Figure 5A).

**Figure 3:**
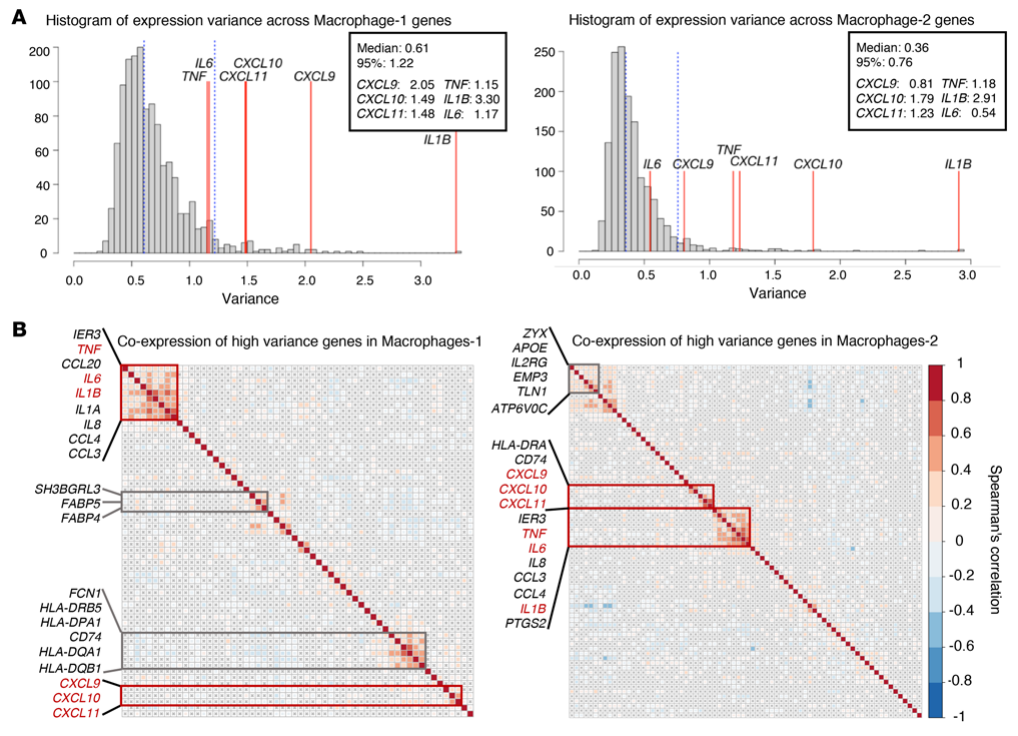
Heterogeneous trained-immunity effect in terms of expression of marker genes among macrophages at T2. (**A**) Distribution of the variation of trained immunity (TI) marker gene expression across macrophages with that of other genes (with expression level log[TP10K + 1] > 0.5). (**B**) Coexpression correlation of TI marker genes and other top 5% high-variance genes in 2 macrophage clusters. TI marker genes are highlighted in red. Red and blue squares in heatmap correspond to significant (Spearman’s *P* < 0.05) positive and negative correlation, respectively; gray cross indicates not significant.

Given the wide range of expression in both monocyte and PBMC groups, we assessed whether the intercell variation of TI signature genes shows larger variation compared with other genes. In both Macrophages-1 and -2 from 4 stimuli conditions (Figure 3A), the 6 TI signature genes showed consistently higher variation across cells compared with that of the other genes expressed at a similar level (i.e., genes with log[TP10K + 1] ≥ 0.5), with the highest level of variation found for *IL1B*. Together, *IL1B*, *TNF*, and *CXCL9-11* are among the top 5% most variable genes, suggesting a heterogeneous TI response among macrophages. To minimize the influence of mean expression values on the variance, we also drew a distribution of the dispersion index (*D* = var/mean) on the same gene sets. Four TI signature genes in Macrophages-1 and all 6 genes in Macrophages-2 can be validated as the highest 5% dispersion index (Supplemental Figure 5B).

We also noticed that these TI signature genes are clustered into 2 groups in both macrophage clusters based on their pair-wise positive correlation patterns with other top 5% most variable genes (Figure 3B and Supplemental Figure 6): the 3 proinflammatory cytokine genes *(IL6*, *TNF*, and *IL1B*) correlated with each other (Spearman’s correlation coefficient, *ρ* > 0.25; *P* < 1 × 10^–6^), and additionally the 3 chemokines (*CXCL9-11*) correlated with each other (Spearman’s correlation coefficient, *ρ* > 0.25; *P* < 1 × 10^–6^).

### Transcriptome analysis reveals diverse subpopulations of TI phenotypes.

**Figure 4:**
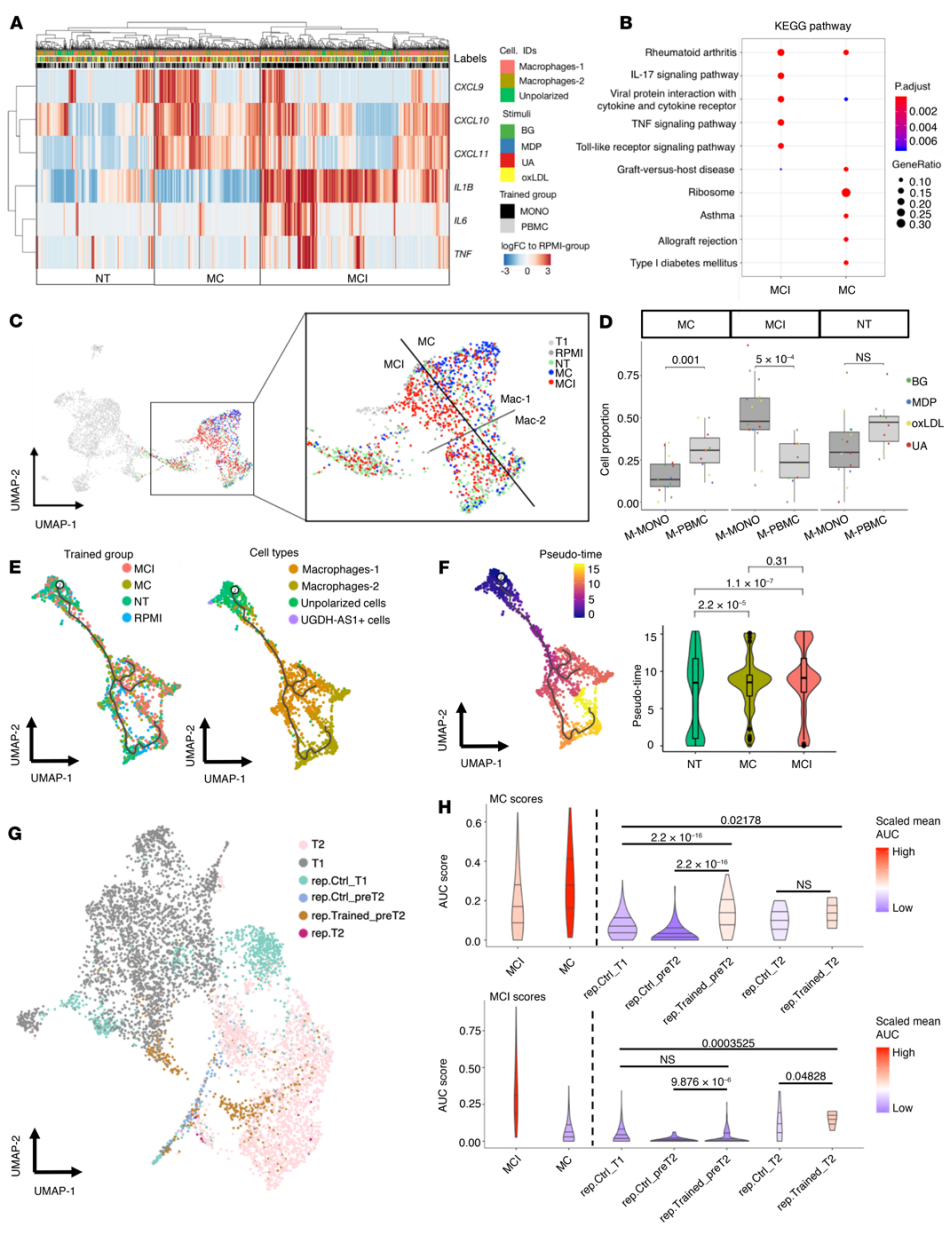
Subgroups of trained cells reveal diverse trained-immunity phenotypes. (**A**) Heatmap showing log(fold change) of 6 marker genes (rows) in trained macrophages relative to the average expression in control macrophages (columns). Red and blue colors correspond to upregulation and downregulation, respectively. (**B**) KEGG enrichment of training response (TR) genes (comparing trained conditions with RPMI controls) in each subgroup of trained cells. (**C**) Annotation of the subgroups of trained cells in UMAP plots. (**D**) Comparison of the cell frequency of subgroups between trained tissues. M-MONO, monocytes trained in the absence of lymphocytes; M-PBMC, monocytes trained in the presence of lymphocytes. Dirichlet’s regression model was applied to test the differences in cell frequency between groups; *P* values are shown on the box-and-whisker plot. (**E**) UMAP of cellular trajectories inferred by Monocle 3 with trained subgroups or original clusters. (**F**) UMAP and violin plot of pseudo-time state of trained cells estimated by Monocle 3. *P* values from Wilcoxon’s rank-sum test are shown on the violin plot. (**G**) Integrated UMAP of cells from the initial and replicate in vitro experiments showing the distribution of cells sampled at different time points. (**H**) Violin plots showing AUCell-based scores (R/AUCell package) of trained-immunity signatures from MCI and MC subgroups in trained cells and nontrained controls sampled from the replicate experiment. The lines in the violin plots represent the median of the AUC scores and the 0.25 and 0.75 quantiles, and colors represent the average scores centered on zero. Wilcoxon’s rank-sum test was applied to ascertain whether the AUC scores in trained cells were larger than in nontrained controls. T1, 4 hours after training (or RPMI) stimulation; pre-T2, 5 days after training (or RPMI) stimulation and before LPS restimulation; T2, 4 hours after LPS restimulation. *P* values are shown at the top in **D**, **F**, and **H**.

TI is characterized by enhanced responses of TI signature markers upon restimulation as compared with untrained cells (7, 8). Thus, at T2, for each cell from the trained conditions, we defined TI phenotypes as expression log(fold change) of TI signature genes relative to their respective average expression in the RPMI-control group upon restimulation with LPS. The initial hypothesis was that potential subpopulations of trained macrophages would display increased production of both proinflammatory cytokines and chemokines, as the production of these mediators during the immune responses is very often simultaneous. However, an unsupervised clustering analysis of TI phenotypes in all macrophages revealed 3 distinct subgroups, depending on the gene expression of chemokines and cytokines (Figure 4A): (a) macrophages with enhanced expression of genes encoding chemokines and proinflammatory cytokines (MCI), as compared with control; (b) macrophages with enhanced expression of chemokines only (MC) as compared with control; and (c) nontrained cells (NT), which are cells with low TI phenotypes. In total, we identified 39.8% MCI, 22.2% MC, and 38.0% NT from T2 macrophages. All 3 subgroups were roughly equally present (no significant difference in the proportion) across all training stimuli, suggesting the phenotypes of identified MCI and MC populations are similar regardless of the stimulation.

In order to understand the potential function of the MCI and MC cells, we performed KEGG pathway enrichment analysis in each subgroup of trained cells. TI response (TR) genes were detected by comparing the gene expression profiles of LPS-restimulated macrophages between TI subgroups and RPMI-control groups (Supplemental Figure 7A and Supplemental Table 2C). TR genes found in the MCI and MC groups were enriched for distinct pathways and disease etiology. In MCI, IL-17 and TNF-α signaling pathways were significantly enriched (*P* < 0.001), while in MC, KEGG pathways associated with asthma and type-1 diabetes mellitus were enriched with several MHC class II genes (including *HLA-DPA1*, *HLA-DQA1*, etc.) (*P* < 0.001; Figure 4B), suggesting a cytokine-signaling-increased function of the MCI subgroup and more potent antigen-presenting function in the MC subgroup. Additionally, GO enrichment analyses revealed that TR genes of MCI and MC were significantly enriched for cellular responses to bacteria and protein targeting to membrane, respectively (*P* < 1 × 10^–6^; Supplemental Figure 7B), implying different functions of these 2 subpopulations in the context of TI.

Interestingly, the 2 identified subgroups of trained cells (MCI and MC) are different from the classic annotation of Macrophages-1 and -2, as shown in the UMAP (Figure 4C). The distribution of MCI cells was clearly independent of Macrophages-1 and -2 polarization (χ^2^ test, *P* = 0.8573), while MC cells significantly overlapped with Macrophages-1 (χ^2^ test, *P* = 2.76 × 10^–6^). This suggests that the identified MCI and MC cells are independent of the macrophage polarization process induced in vitro.

### The effects of the presence and absence of lymphocytes.

We investigated whether the presence of lymphocytes during the first 24 hours of the TI experiment (M-PBMC group) resulted in a different program compared with monocytes that were trained without the presence of lymphocytes (M-MONO group). First, we compared the relative abundance of MCI and MC cells in these 2 groups (Figure 4D and Supplemental Figure 7C). Interestingly, for all training conditions, the population of MCI cells was more abundant in the M-MONO group as compared with the M-PBMC group (Dirichlet’s regression test, *P* = 5 × 10^–4^). In contrast, the population of MC cells was significantly higher in the M-PBMC group (Dirichlet’s regression test, *P* = 0.001), suggesting that monocytes in the PBMC environment have a higher potential to be trained as MC regardless of the training stimulus. These findings are in line with the observation of stronger TI-increased CXCL10/CXCR3 responses in the presence of T cells than without (8).

To characterize the transcriptional differences within the trained subsets, we investigated DEGs by comparing cells from the M-PBMC and M-MONO groups in both MCI and MC subsets of trained cells. From the results (Supplemental Figure 8A and Supplemental Table 2D), we identified several genes, such as *IL6* (*P_adj_* = 8.67 × 10^–6^), *IL1B* (*P_adj_* = 3.58 × 10^–3^), and *CXCL3* (*P_adj_* = 8.96 × 10^–5^) in MCI cells as well as *CD74* (*P_adj_* = 9.18 × 10^–3^) and *HLA-DQA1* (*P_adj_* = 2.26 × 10^–2^) in MC cells that stand out from the M-MONO group, whereas *SERPINB2* (*P_adj_* = 1.72 × 10^–12^ in MCI and 5*.*37 × 10^–10^ in MC), *CEBPB* (*P_adj_* = 1.11 × 10^–4^ in MCI and 8.31 × 10^–6^ in MC), and *HLA-B* (*P_adj_* = 1.99 × 10^–5^ in MCI and 4.51 × 10^–6^ in MC) show consistently higher expression in both subsets of the M-PBMC group. Although most of the changes were minor (log[fold change] < 0.5), in the KEGG enrichment of these DEGs, the IL-17 signaling pathway was found in M-MONO groups in MCI (Supplemental Figure 8B). However, the enriched genes, *IL6*, *IL1B*, *CXCL3*, and *RELA*, were not specific to IL-17 and other genes in the IL-17 pathway were not significantly changed. The results suggest no major differences in M-PBMC versus M-MONO with regard to transcriptional responses or signaling pathways in each trained subset.

To further address the lymphocytes’ effects on transcriptional regulation at the priming stage, we investigated the DEGs by comparing monocytes under training between the M-PBMC and M-MONO groups at T1 (Supplemental Figure 8C and Supplemental Table 2D). In total, 28 genes were found significantly upregulated in the M-PBMC group, which included *NFKBIA*, *JUN*, *FOSB*, *CXCL2*, *CXCL3*, and *CCL4* that were subsequently enriched in the IL-17 and NF-κB signaling pathways. On the other hand, 82 genes were found significantly upregulated in the M-MONO group, including *CCL3*, *IL1B*, and several *MHC* class II genes that were enriched in autoimmune disease pathways (such as rheumatoid arthritis and asthma) (Supplemental Figure 8D). The results indicate that lymphocytes influenced the transcriptional responses of monocytes to training, which might be related to the observed differences in the relative abundance of the trained subsets.

### Two subpopulations of trained cells follow a different cellular trajectory.

In response to stimulation, cells transit from one functional state to another; thus, 2 cell subpopulations could be just 2 states of 1 transition process. Cellular trajectory analysis was introduced to scRNA-seq to order the captured cells along a reconstructed trajectory of cellular transition and estimate a pseudo-time state for each cell. In order to test whether the subpopulations of MCI and MC trained cells with distinct TI phenotypes follow the sequential states of 1 linear trajectory or not, we applied Monocle 3 (22) to obtain fully unsupervised estimates of the cellular trajectories and pseudo-time states among all the T2 cells. As shown in Figure 4E, the cell trajectory started from the unpolarized cells and subsequently divided into 3 branches, 2 of which derived toward Macrophages-1, and 1 further derived toward Macrophages-2. Most of the MCI and MC cells were located at the end tips of those branches, while cells from the NT groups and RPMI control were scattered along the whole trajectories. This indicates that MCI and MC were 2 distinct subgroups and were both in the late stages along the trajectory of captured cells, while cells from the NT groups and RPMI control had mixed time stages.

As shown in Figure 4F, a similar conclusion could be drawn from the pseudo-time analysis (22). The unpolarized cells were assigned as the beginning of the trajectory (time = 0), whereas the MCI and MC cells were both allocated to a similarly late time state (time = 7–12). Student’s *t* test showed that the MCI and MC groups were significantly older than NT cells in terms of estimated pseudo time of each cell, with *P* = 1.1 × 10^–7^ and 2.2 × 10^–5^, respectively. However, the estimated pseudo time of MCI and MC cells showed no significant difference (*P* = 0.31), suggesting that there was no transitional order between the MCI and MC groups. To sum up, MCI and MC cells differ in both TI phenotypes and transcriptomic profile.

### Transcriptome profiles of trained cells prior to restimulation.

To further distinguish the observed heterogeneous TI effects with transcriptional changes induced by incubation, we performed an independent in vitro replicate study with identical settings and obtained 13 samples from cell-multiplexed scRNA-seq, including 8 samples from incubated trained/untrained cells prior to restimulation (pre-T2), 3 nonincubated monocyte samples (T1), and 2 LPS-restimulated macrophages (T2) (see Methods, Supplemental Figure 9A, and Supplemental Table 1B for details). With an integrated visualization with all in vitro samples, all the monocytes/macrophages prior to restimulation (pre-T2) distributed between T1 and T2 cells (Figure 4G), indicating that the 5-day incubation shifted the transcriptomics of both trained and nontrained monocytes. In order to assess whether the TI signatures of the MCI/MC subgroups identified from the previous experiment can also be detected in the data before restimulation, we applied the AUCell-based (24) enrichment scoring method to these validation samples. Compared with the nontrained cells (T1), the MCI scores of trained cells prior to restimulation show no differences (Wilcoxon’s rank-sum test, *P* = 1), but the MCI scores of cells after LPS restimulation show significantly (Wilcoxon’s rank-sum test, *P* = 0.02178) higher scores (Figure 4H). In contrast, the MC scores of trained cells from both before and after restimulation show significantly higher MC scores compared with those of nontrained T1 cells. These results indicate that the MCI cells were more specifically responding to restimulation, and the MC cells might have been differentiated already during incubation. Moreover, since both scores from nontrained controls are lower after 5 days of incubation (pre-T2) than before (T1), this further indicates that the activation of both trained subsets comes from training stimulation instead of incubation.

### Shared and specific transcriptional programs in cells trained by different stimuli.

**Figure 5:**
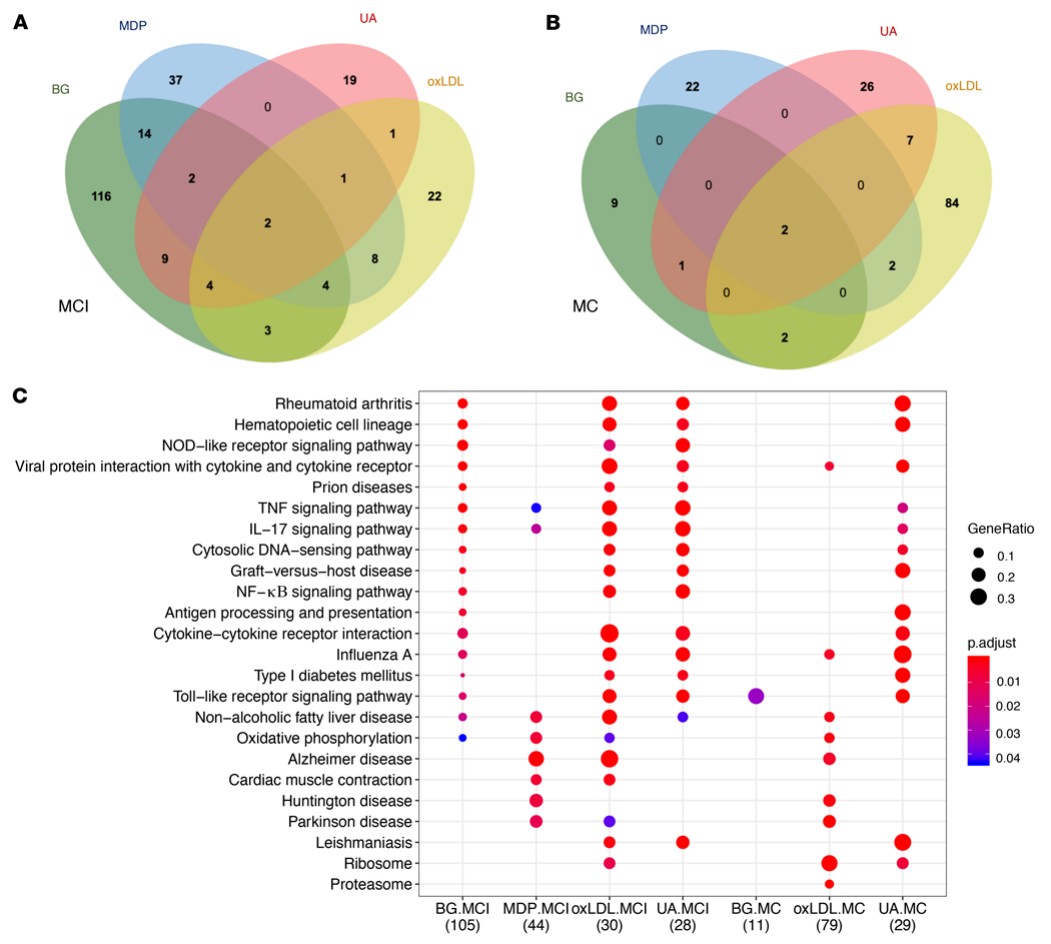
Gene signatures found in trained subgroups. (**A**) Venn diagram showing the number of training response (TR) genes found in MCI (**A**) and MC (**B**) across the different inducers of trained immunity. (**B**) Dot plot showing the KEGG enrichment of TR genes found in MCI/MC across the different inducers of trained immunity.

In order to identify the shared and specific set of TR genes in the cells trained by different stimuli, we performed the TR analysis for each stimulation condition (Supplemental Table 2E). A summary of all identified TR genes across conditions is illustrated in Figure 5, A and B. In terms of genes, we observed 48 genes shared by at least 2 training stimuli in MCI cells and 14 genes from MC cells (Supplemental Table 2E). Among them, however, only *PTGS2* and *IL1B* were upregulated in all stimuli (*P_adj_* < 1 × 10^–4^ in each condition) from the MCI group, and *CXCL10*/*11* were upregulated in all stimuli (*P_adj_* < 0.05 in each condition) from the MC group. In terms of pathway enrichment, these TR genes revealed that the TI effects were shared across different stimuli for the MCI group, but they showed specificity in the MC groups. In MCI groups, TR genes from different stimuli conditions were consistently enriched in NOD-like receptor signaling pathway, IL-17 and TNF-α signaling pathways, as well as cytokine-cytokine receptor interaction (Figure 5, A and C). In contrast with the MCI group, few numbers of DEGs or KEGG terms were found to be shared across different stimuli in the MC group (Figure 5, B and C). KEGG pathway analysis in the MC group revealed that DEGs induced by UA were enriched in asthma and allograft rejection, while Parkinson disease and oxidative phosphorylation were enriched only in the oxLDL-trained group, suggesting the cellular responses of those stimuli are different (Figure 5C).

### TI signatures in patients with immune diseases.

**Figure 6:**
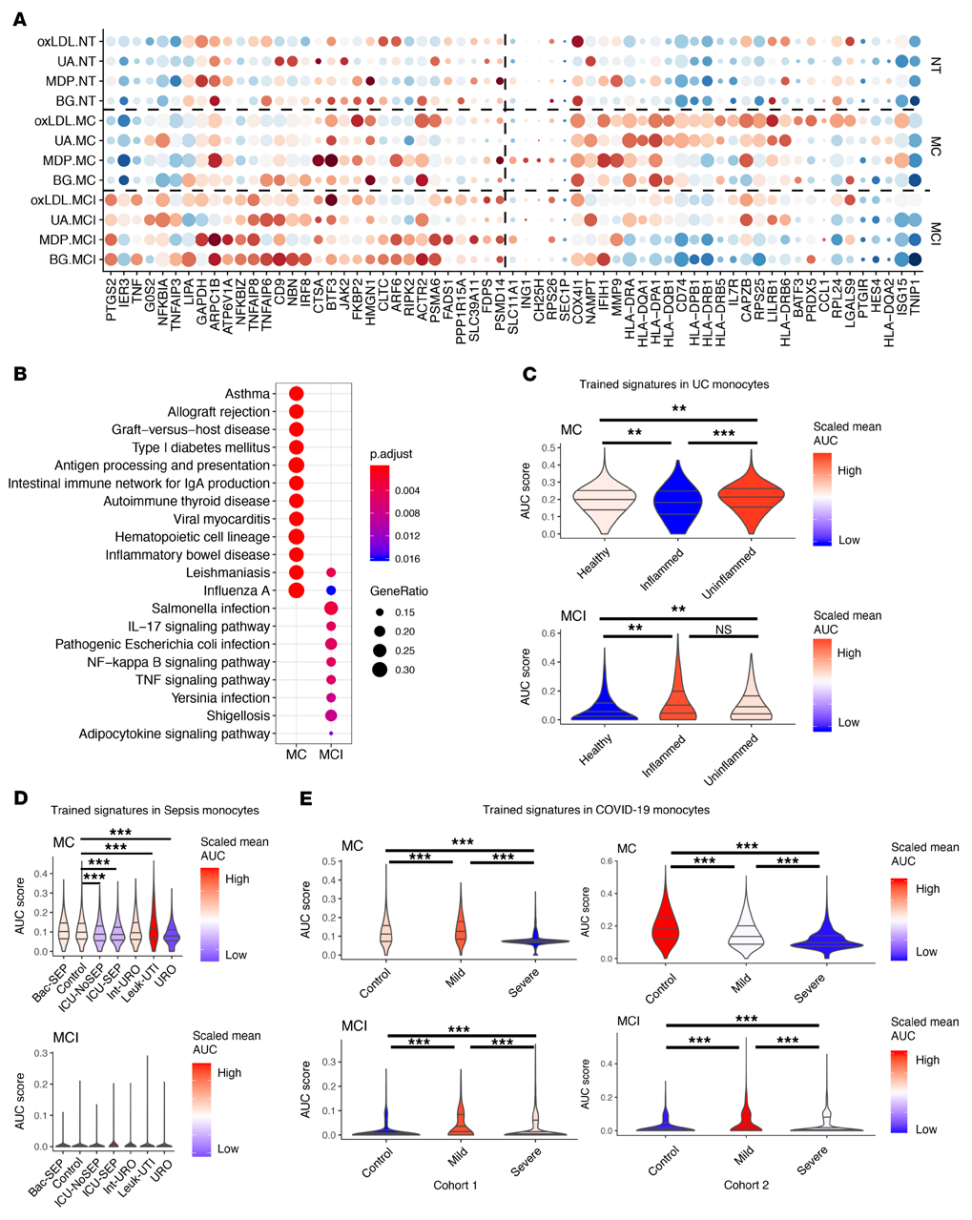
Expression of trained-immunity signatures in infectious diseases. (**A**) Dot heatmap of training response (TR) genes around GWAS-risk loci of inflammatory bowel disease (IBD). DEGs found in MCI/MC across different stimuli were enriched in genes 250 kbp around GWAS-risk loci of IBD in comparison to genes 250 kbp around height-associated loci (Fisher’s exact test, *P =* 0.0025). (**B**) KEGG enrichment of TR genes around GWAS-risk loci of IBD/ulcerative colitis (UC). (**C–E**) Trained-immunity signatures in monocyte clusters of patients with UC (**C**), sepsis (**D**), and COVID-19 (**E**). The scRNA-seq data sets used for panels C–E are from Smillie et al. (25), Reyes et al. (26), and Schulte-Schrepping et al. (27), respectively. The lines in the violin plots represent the median of the respective AUC scores (R/AUCell package) and the 0.25 and 0.75 quantiles, while colors in the violin plots represent the average AUC scores centered on zero. Wilcoxon’s rank-sum test was applied to compare the AUC scores between clinical conditions recorded in each study. **P < 1 × 10^–5^; ***P < 1 × 10^–10^.

Additionally, we tested whether the TR genes overlapped with disease-associated genes identified in genome-wide association studies (GWAS), including inflammatory bowel disease (IBD), ulcerative colitis (UC), and cardiovascular disease. Interestingly, 63 genes within 250 kbp of GWAS risk loci reported in studies performed in IBD/UC patients (23) also responded to training in either the MCI or MC group (Figure 6, A and B; Fisher’s exact test, genes within 250 kbp of height-associated SNPs were used as reference trait, *P* = 0.0025). Additionally, 32 TR genes (including *GBP1*, *IFI30*, *CSTB*, *ACTR2*, etc.) were found within risk loci of cardiovascular disease (Supplemental Figure 9B; Fisher’s exact test, *P* = 0.394). More interestingly, among the risk genes associated with IBD/UC, we observed that some genes (such as *PTGS2*, *TNFAIP8*, *TNFAIP6*, and *CD9*) were mostly upregulated in the MCI group, while other genes (such as *HLA-DR*, *CD74*, *IFIH1*, and *ISG15*) were only found upregulated in the MC group. KEGG enrichment analyses showed that these IBD/UC-risk genes from the MC group were enriched in intestinal immune network for IgA production and autoimmune thyroid disease, while the IBD/UC-risk genes from the MCI group were enriched in TNF/IL-17 signaling pathways (Figure 6B). These findings suggest a potential role for TI in IBD/UC and that both subgroups might contribute to the pathology.

Next, we assessed whether the signature from the MCI/MC subgroups can also be detected in patients with infectious diseases. To do so, we assigned AUCell-based enrichment (24) of MCI/MC signature scores to monocytes and macrophages from recently published scRNA-seq data sets from patients with UC (25), sepsis (26), and COVID-19 (27). First, we identified MCI/MC signatures by DE analyses for upregulated genes between cells in each trained group and the rest of cells. In total, 9 MCI signatures (*IL1B*, *IL8*, *IL6*, *PTGS2*, *IL1A*, *CCL2*, *TNF*, *CXCL3*, and *CXCL1*) and 12 MC signatures (*CXCL11*, *CXCL10*, *CXCL9*, *TNFSF10*, *HLA-DQA1*, *FCN1*, *IGFBP4*, *HLA-DPB1*, *HLA-DQB1*, *FAM26F*, *RGL1*, and *CD4*) were obtained and subsequently used to calculate a signature score for each monocyte in the scRNA-seq data sets of patients.

MC signatures were found in monocytes of both patients and healthy controls, whereas MCI signatures were mostly found in patients. In the UC study (25), monocytes from both inflamed and uninflamed tissues of UC patients showed higher MCI signature scores than healthy controls (Wilcoxon’s test, *P =* 1.88 × 10^–9^ and 3.83 × 10^–9^), whereas no significant differences were found between inflamed and uninflamed tissue from patients (Figure 6C and Supplemental Figure 9C). On the other hand, inflamed tissue from patients showed lower MC signature scores than uninflamed tissue and healthy controls (Wilcoxon’s test, *P =* 2.50 × 10^–15^ and 5.38 × 10^–7^). This might suggest that MCI-associated genes are globally activated in monocytes of UC patients, but MC signatures are potentially suppressed in the UC inflammatory responses.

In monocytes from sepsis patients (26), we identified MC signatures that were higher in milder patients (Leuk-UTI) but lower in more severe patients (ICU-SEP, ICU-NoSEP, and URO) (Wilcoxon’s test, *P* < 2.2 × 10^–16^; Figure 6D and Supplemental Figure 9D), which indicates that MC signatures might be suppressed in severe patients. In monocytes from COVID-19 patients (27), we found significantly higher MC and MCI signatures in mild patients compared with severe patients (Wilcoxon’s test, *P* < 2.2 × 10^–16^; Figure 6E and Supplemental Figure 9E), which again demonstrated that severe patients have suppressed TI signatures (28).

### In vivo validation of the identified TI subpopulations.

**Figure 7:**
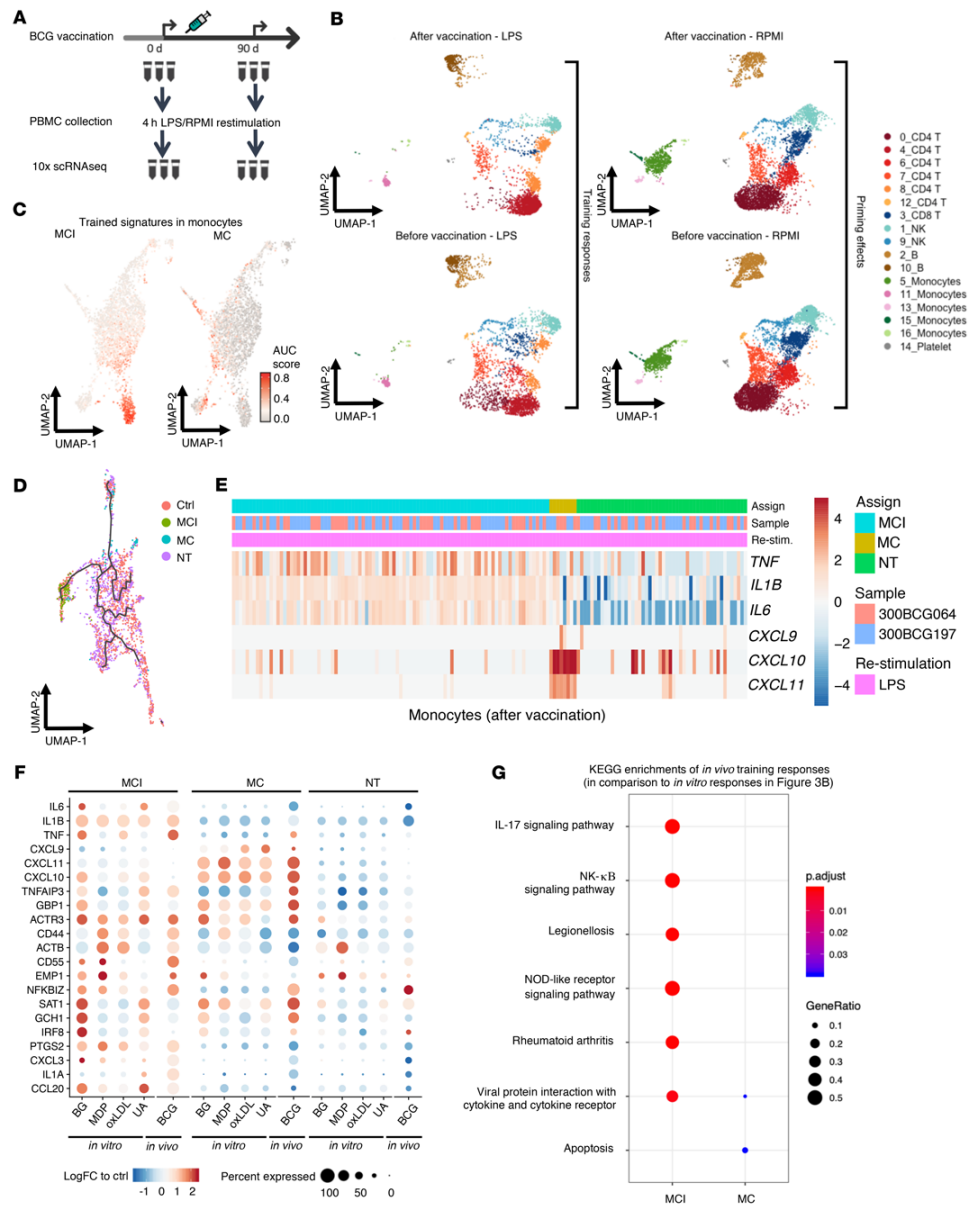
In vivo validation of trained-immunity signatures. (**A**) Study design. Healthy human volunteers (*n =* 3) were vaccinated with BCG. Before vaccination and 90 days later, PBMCs were isolated and restimulated ex vivo with RPMI culture medium (control) or LPS. (**B**) UMAP of PBMCs showing cells before and after BCG vaccination, with or without LPS restimulation from the in vivo study. (**C**) UMAP of AUC scores in monocytes after BCG vaccination. (**D**) UMAP of cell trajectory of monocytes after BCG vaccination, annotated by assigned trained subgroups. (**E**) Heatmap showing log(fold change) of 6 marker genes (rows) in monocytes 90 days after BCG vaccination (column) relative to the average expression before vaccination. Red and blue colors correspond to upregulation and downregulation, respectively. (**F**) Dot heatmap of expression of shared training response (TR) genes detected in LPS-restimulated cells from trained subgroups in both in vivo and in vitro training experiments. Gene expression is shown as log(fold change) relative to the average of RPMI control groups in the in vitro study, and relative to time point before vaccination for the in vivo study. (**G**) KEGG enrichment of TR genes of the in vivo study in each subgroup of trained cells.

To validate the identified TI signatures of MCI and MC in vivo, we applied 10× Genomics scRNA-seq on PBMCs isolated from 3 healthy donors before and 3 months after BCG vaccination with and without LPS restimulation (Figure 7A). In total, 17 clusters were identified in 6,872 restimulated PBMCs and 12,717 nonrestimulated PBMCs from all samples, in which 5 clusters were annotated as monocytes based on their expression of marker genes (Figure 7B, Supplemental Figure 10, and Supplemental Table 2F). To explore the BCG-induced TI phenotypes, we applied the above-mentioned AUC-based signature scores to align MCI and MC signatures from in vitro data with the BCG-vaccinated monocytes. In total, 93 and 8 LPS-restimulated monocytes were assigned as MCI and MC cells, respectively (Figure 7C), suggesting a different ratio of MCI/MC subgroups compared with in vitro data. A trajectory inference with trained subgroups and other untrained monocytes showed that the MCI and MC subgroups followed 2 discrete trajectories derived from untrained monocytes (Figure 7D), which is consistent with our findings in the in vitro–trained cells (Figure 4, C and E).

After assigning the in vivo–trained subgroups, we assessed their TR genes by comparing gene expression profiles of LPS-restimulated monocytes after BCG vaccination to the levels before vaccination. Log(fold change) of 6 TI markers showed a similar pattern to that observed in in vitro–trained cells. Overall, the MCI subgroup showed higher transcriptional responses of proinflammatory cytokines and slightly higher responses of chemokines, while the MC subgroup showed much higher responses of chemokines (Figure 7E). Among the 48 TR genes found in shared conditions within in vitro–trained subgroups, 21 of them could be replicated in the in vivo–trained cells (Figure 7F and Supplemental Table 2G). In the KEGG enrichment analyses of significant TR genes in each subgroup of trained cells, the IL-17 signaling pathway and pathway associated with rheumatoid arthritis were significantly enriched in MCI, while in MC, the TR genes were significantly enriched in apoptosis (Figure 7G). This is again in agreement with the in vitro data (Figure 4B), suggesting the robustness of the defined MCI and MC populations.

To compare the TR genes with the transcriptional alteration caused by BCG vaccination, we compared BCG-trained and untrained monocytes (before BCG vaccination), without LPS restimulation. Upon BCG vaccination, 57 genes were upregulated and 56 downregulated, all of which showed minor changes in gene expression (only 2 genes had log[fold change] > 1: *CCL3* and *CCL4*; Supplemental Table 2H). Among the upregulated genes, only 4 of them (*IL1B*, *DNAAF1*, *CD55*, and *CXCL8*) were also TR genes that were upregulated in trained monocytes upon LPS restimulation (Supplemental Figure 11A). Other genes that responded to TI, such as *CXCL9-11* chemokines, *TNF*, and *IL6*, as well as other potential TI signatures such as *CXCL2*, *CXCL8*, *GBP1*, and *PTGS2*, did not significantly differentially respond to BCG vaccination without LPS restimulation. The GO and KEGG enrichments of these nonrestimulated DEGs showed few enriched terms but only ribosome and protein targeting to ER in upregulated genes after BCG vaccination (Supplemental Figure 11, B and C). The enriched terms found in trained groups, such as IL-17 and NF-κB signaling pathways, were either not enriched or enriched in downregulated genes. Together, these results indicate that trained monocytes are epigenetically primed to transcriptionally differently respond to a secondary stimulus, but that in the resting state they do not display major changes in their transcriptional program.

**Figure 8:**
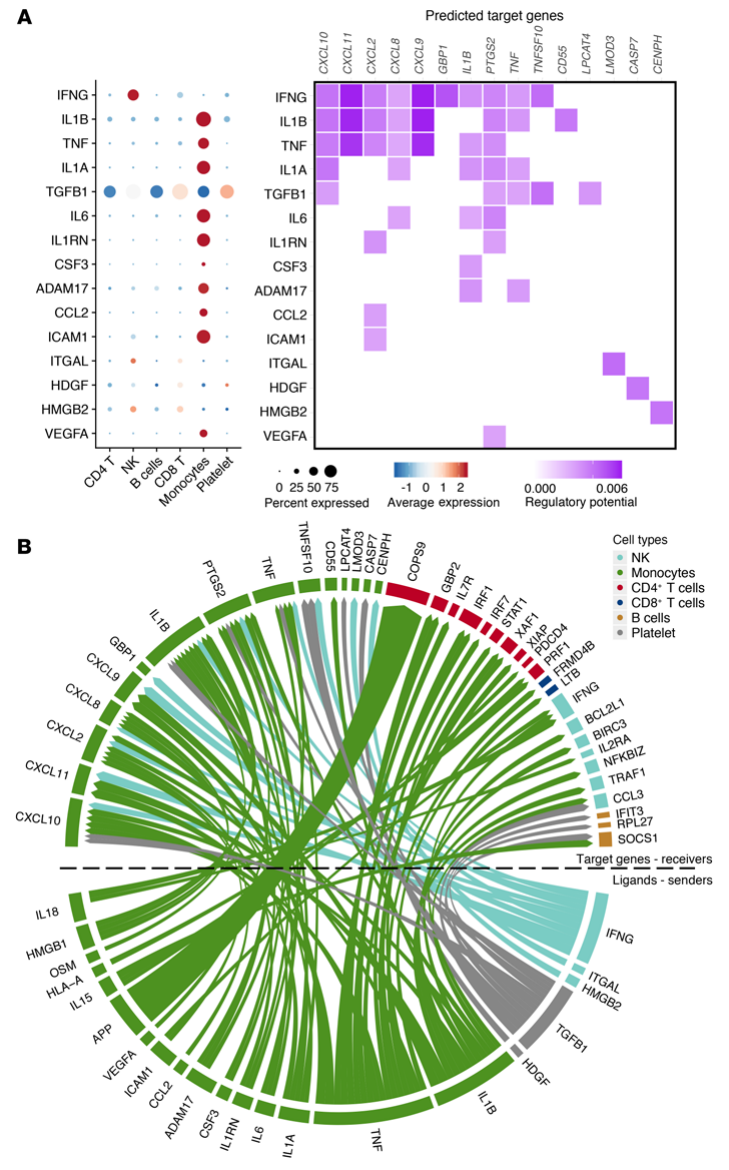
Cell-cell interaction in inducing the trained-immunity transcriptional responses. (**A**) Dot heatmap of the top 15 predicted ligands and heatmap of respective target genes regulated by top-ranked ligands. (**B**) A circos plot shows the predicted top ligands from sender cells and their target genes from different receiver cells.

Next, we tested how cellular interactions that were responsible for inducing the TI transcriptional responses of MCI and MC subgroups by applying NicheNet (29). Interestingly, most top-ranked ligand genes were expressed in the monocyte compartment, while IFN-γ expressed in NK cells and TGF-β1 expressed in CD8^+^ T cells were also predicted as the top 1 and top 5 ligands, respectively (Figure 8A). TI markers (CXCL9-11 chemokines, TNF-α, and IL-1β) were predicted as target genes of top-ranked ligands. In addition, *CXCL2*, *CXCL8*, *GBP1*, *PTGS2*, *TNFSF10*, and *CD55* were also predicted as target genes of those ligands, suggesting them to be novel TI signature candidates. To determine the effect of trained monocytes on other immune cells in the niche, we then assigned the trained monocytes as senders and predicted their effects on the gene expression of other immune cells. As shown in the circos plot (Figure 8B), in addition to receiving signals, the trained monocytes were also sending ligands to affect the expression of genes such as *GBP2*, *IL7R*, and *IFNG* in other immune cells, including NK and CD8^+^ T cells. Interestingly, we observed a potential regulation pathway in which higher expression of *IL1B* and *IL18* in MCI subpopulations induced the expression of *IFNG* in NK cells, which then enhanced TR genes such as *TNF*, *PTGS2*, and *CXCL9*-*10* chemokines. Thus, the results suggest that one trained cell population might induce changes in another population, which has also been reported, for example, in neutrophils upon helminth infection, which led to a TI phenotype in macrophages (30).

## Discussion

The highly divergent transcriptional responses of cytokine and chemokine genes have been reported as an important feature of innate immune cells across different species, and the regulation on the distinctive promoter architecture of these genes appears to be related to their high divergence (31). Our study also reveals a large heterogeneous response of TI signature cytokines and chemokines among trained monocytes/macrophages upon LPS restimulation at the single-cell-transcriptome level. We subsequently reclustered training-induced monocytes/macrophages into MCI, MC, and NT subpopulations, which were characterized by high transcription levels of cytokines and chemokines or not (nontrained cells). We demonstrated pathway enrichments that suggest functional differences of 2 well-trained subpopulations (MCI and MC). To our best knowledge, the present study is the first to describe cellular heterogeneity of TI at the single-cell-transcriptome level.

Our results show that monocytes consistently differentiated into 2 macrophage clusters after 5 days of in vitro incubation, regardless of previous exposure to a TI stimulus. This consistency was further supported by the statistical test on the cell proportion of each cluster, where no significant change was observed between any 2 stimulus conditions and control. The TI characteristics were induced in well-trained subpopulations in both Macrophages-1 and -2, where we found more DEGs compared with the RPMI-control group with BG than in the other 3 conditions. This indicates that BG performed as the strongest training stimulus in our study. Noticeably, significant upregulated *IL1B* expression was shown after direct stimulation by BG, supporting earlier findings that indicate that IL-1β could also induce TI in monocytes (32).

Another important aspect to investigate is whether the intercellular interactions in blood and tissues influence the TI process. Previous evidence has suggested that CXCL10 chemokine production by monocytes requires the presence of CXCR3^+^ T cells, while IFN-γ production by NK cells will also affect BCG-induced TI responses (33). In our in vitro study, we trained monocytes in the presence (M-PBMC) and absence (M-MONO) of lymphocytes, but considering the limitation of cells numbers and replicates in our data set and the similarity shown in 2 groups (Figure 2B and Supplemental Figure 2), we joined 2 groups to increase statistical power in discovery stages and validate their differences in detailed analysis. Interestingly, we observed a significantly higher percentage of MC subpopulations in samples from the M-PBMC group. This suggests that the T cell presence during induction of TI amplifies the induction of TI, as mirrored by the higher expression of genes encoding CXCL9-11 chemokines in the monocytes trained in the presence of lymphocytes (in the PBMC model), compared with the purified monocytes. On the other hand, in our PBMC samples from BCG-vaccinated individuals, although we identified both MCI and MC subpopulations, we observed a much higher percentage of the MCI subpopulation and lower MC subpopulation, compared with every condition of the in vitro samples. These results imply that more complex cellular interactions of the in vivo environment mediate the TI process. Therefore, further investigation on the intercellular interactions of TI processes with more abundant in vivo data is warranted.

Reprogramming of innate immune cells could play a beneficial or deleterious role in infectious diseases and/or autoimmune diseases (34). In both IBDs, Crohn’s diseases (CD) and ulcerative colitis (UC), the patient’s immune system attacks elements of the digestive system. In our study, we have found genes around GWAS risk loci of CD/UC patients significantly enriched in DEGs detected in MCI/MC subpopulations. Those genes again formed distinct subgroups as upregulated in either MCI or MC in our study. Moreover, MCI signatures were also found significantly higher in both inflamed and uninflamed tissue of UC patients than tissue in healthy individuals. This indicates that the trained monocytes/macrophages could play a role in immune responses of IBD/UC patients (25). In addition, dysfunction of innate immune responses of monocytes as has been described in sepsis (26), and COVID-19 (27) also contributes to the pathology of the disease. In our study, we observed significantly reduced TI signatures in monocytes from severe sepsis patients (reduced MC signatures) and COVID-19 patients (reduced both MCI/MC signatures). Since the MCI/MC signatures in our study were defined as enhanced responses instead of novel responding signatures, our results demonstrated a negative correlation between enhanced signatures and severity. This indicates that the enhanced proinflammatory responses associated with TI might help to fight certain infections; this hypothesis has indeed been extensively studied in the COVID-19 pandemic, with more than 15 clinical trials now investigating the capacity of BCG vaccination to decrease the severity of COVID-19 (28). Our findings also indicate that both identified subpopulations of trained cells could play important roles in these conditions and highlight the importance of TI programs in mediating pathogenetic mechanisms in infections and inflammatory diseases, and suggest therapeutic and/or prevention implications that need to be investigated in further studies.

Collectively, our dual scRNA-seq study with in vitro and in vivo samples provides robust and reproducible results concerning the heterogeneity of subpopulations of TI transcriptional responses in monocytes and macrophages. Our study is relatively limited by the sample size and sequencing depth and likely did not capture all transcriptional signals of cell surface markers or cytokine/chemokine markers of different subpopulations, and future studies are warranted. In future studies, it will be important to systematically dissect the subpopulations at multiple layers of regulation, such as a hybrid single-cell study at both the transcriptomic and epigenomic level. Additional in vivo studies of TI with larger sample sizes and higher resolution would also be important for deeper investigation of this nonspecific adaptive characteristic of innate immune cells. Finally, the functional consequences of these subpopulations of monocytes should be investigated in response to various pathogens, in order to obtain a comprehensive understanding of their function.

## Methods

### Sample collection and cell sorting.

Isolation of primary cells and the in vitro model of TI was performed as described previously (10). Briefly, PBMCs from 3 donors were isolated by density centrifugation in Ficoll-Paque (GE Healthcare), washed twice in PBS, and resuspended in RPMI culture medium (Invitrogen) supplemented with 50 μg/mL gentamicin, 2 mM Glutamax (GIBCO), and 1 mM pyruvate (GIBCO). In the M-PBMC group, PBMCs were incubated with culture medium only (negative control), 2 μg/mL BG, 500 μg/mL UA, 10 μg/mL oxLDL, or 10 μg/mL MDP for 24 hours. Cells were washed with PBS and incubated for 5 days in culture medium supplemented with 10% pooled human serum. On day 6, cells were restimulated with 10 ng/mL LPS for 4 hours (Figure 1A). In the M-MONO group, the same experiment was performed using Percoll-isolated monocytes.

### scRNA-seq.

To more efficiently capture single-cell transcriptomes of TI, we sorted monocytes from all samples based on forward and side scatter 4 hours after the first stimulation and 4 hours after restimulation on day 6, and performed well-based scRNA-seq using the SORT-seq platform (19) (a sorting and robot-assisted sequencing based on the CEL-Seq2 protocol). Living single cells from each donor and each stimulus were sorted into 192- or 384-well plates and the transcriptome of each well containing a single cell was sequenced. In total, 60 samples (3 donors × 5 stimuli × 2 conditions × 2 time points) were loaded into 43 plates, with 1 to 4 samples per plate. These plates were sequenced in 2 batches in which conditions and time points were mixed to minimize potential batch effects (for details see Supplemental Table 1A).

### Reads processing and quality control of in vitro study.

Paired-end reads were first processed with the SORT-seq pipeline. Fastq reads were aligned to the human transcriptome (GRCh38) with BWA (35). Read 1 was used for assigning reads to correct cells and libraries, while read 2 was mapped to reference. Duplicated reads with identical barcode or reads that mapped equally to multiple locations were discarded. The output of this pipeline is a digital gene expression (DGE) matrix for each sample, which records the number of unique molecular identifiers (UMIs) for each gene (including protein-coding genes, mitochondrion genes [MT genes], and External RNA Controls Consortium [ERCC] spike-ins) that are associated with each cell barcode. Rare genes detected in fewer than 3 cells were first removed from the DGE matrix, and then low-quality cells were further filtered based on the following strategy: (a) cells with number of detected genes less than 100 or more than 7,000 were removed to avoid empty wells or doublets; (b) cells with more than 25% of MT-gene counts or more than 90% of ERCC spike-in counts were also removed to avoid dying or damaged-membrane cells.

### Data integration and clustering of the in vitro study.

The Seurat v3.1 integration workflow with SCTransform normalization method (36) was used to cluster cells from different samples into distinct cell subsets. We followed this workflow with the following steps: First, we SCTransformed each sample and merged them within each stimulation group (4 training groups and 1 control). Next, we selected 2,000 variable features among 5 data sets and identified anchors from these features to integrate the data sets. These 2 steps corrected batch effects and prevented cell clustering by donor or stimulation phenotypes rather than by cell type or cell subset. Principal component analysis (PCA) was then performed on the integrated data sets, followed by shared nearest neighbor (SNN) graph construction using PC1 to 20 and *k* = 20 nearest neighbors to identify unsupervised cell clusters. Finally, UMAP was used to visualize the cell clusters.

In order to preserve the biological differences for downstream analyses, the above-mentioned batch correction was only used in the cell clustering- and PCA-related steps. For the other analyses, we used standard log-normalization methods, and the original gene counts for each cell were normalized by total UMI counts and multiplied by 10,000 (TP10K), and then log transformed by log(TP10K + 1).

### DEGs.

DEGs were estimated using the FindMarkers/FindAllMarkers functions in Seurat v3.1 with Wilcoxon’s rank-sum test. For each comparison, unless specifically described, genes with at least 10% expression in the tested group and Bonferroni-corrected *P* values less than 0.05 were regarded as significantly DEGs. Cluster or subgroup marker genes were identified by applying DE tests for upregulated genes between cells in one cluster/subgroup to all other clusters in the tested data set.

TR genes were defined as genes detected by DE analysis between LPS-restimulated macrophages of trained subgroups (primed by BG, UA, oxLDL, or MDP) versus LPS-restimulated macrophages of RPMI controls (primed by RPMI) in the in vitro experiments. In the in vivo study, TR genes were defined as genes detected by DE analysis between LPS-restimulated monocytes of trained subgroups (cells collected 3 months after BCG vaccination) versus LPS-restimulated macrophages of NT groups (cells collected before BCG vaccination). Here, training was induced by BCG vaccination.

### Cell type (cluster) annotation.

In order to annotate a meaningful biological cell identity to each cluster, we used a double-checking strategy for the inference (37) by comparing data-derived marker genes with public databases, and by directly visualizing the expression pattern of literature-derived marker genes. Data-derived marker genes were first found by applying DE tests between cells in one cluster and all other cells in the data set. Cluster marker genes were then compared with their reported cell types in human PBMCs in CellMarker databases (38). Then, genes and cell surface markers reported in other monocytes or macrophages analyses were regarded as literature-derived markers, and we visualized their expression levels in each of the identified cell clusters to manually check the cell identities.

At T1, the monocytes clusters were annotated with the following gene signatures: classical monocytes (*CD14*^+^, *CCL2*^+^, *S100A9*^+^, *FCGR3A*^–^, and *CD86*^–^), intermediate monocytes (*CD14*^+^, *FCGR3A*^+^, and *HLA-DRA*^+^), nonclassical monocytes (*CD14*^lo^, *FCGR3A*^+^, *VMO1*^+^, and *HLA-DRA*^+^), and monocytic dendritic cells (*CD14*^–^, *FCGR3A*^–^, *CD86*^+^, and *HLA-DRA*^++^). At T2, the macrophages were annotated with the following gene signatures: Macrophages-1 (*CD14*^–^, *FCGR3A*^–^, *HLA-DRA*^++^, *CD36*^+^, *CD83*^+^, *CD68*^+^, *IL1B*^+^, *IFITM1*^+^, *MSR1*^–^, *SPP1*^–^, *ITGAM*^–^, and *LPL*^–^) and Macrophages-2 (*CD14*^–^, *FCGR3A*^–^, *HLA-DRA*^lo^, *CD36*^+^, *CD83*^+^, *CD68*^+^, *IL1RN*^+^, *MSR1*^+^, *SPP1*^+^, *ITGAM*^+^, and *LPL*^+^).

Two subpopulations detected at T1 were specific to the training experiment performed in PBMCs. One of the expressed genes was enriched in glycolysis and HIF-1 signaling pathways, whereas the marker genes from the other subpopulation were enriched in estrogen signaling and antigen processing and presentation pathways (Supplemental Figure 3B), suggesting their functions in metabolism and signaling processing during stimulation. Thus, these 2 subpopulations are labeled as “HIF-1 signaling cells” and “antigen-signaling cells,” respectively. In addition, we identified a small cluster with high expression of several long noncoding RNA genes (e.g., *UGDH-AS1*, *KCNQ1OT1*). This cluster presented mostly at T1 in all conditions but no known marker genes were detected. Thus, it was labeled as “UGDH-AS1” cells. Cluster-specific marker genes from this cluster were enriched in epidermal growth factor receptor tyrosine kinase inhibitor resistance and vascular endothelial growth factor signaling pathway.

### KEGG and GO enrichment analyses.

R package clusterProfiler v3.10.1 (39) was used for KEGG and GO enrichment analysis for overrepresented pathways and GO terms with DEGs found for each trained group as well as DEGs around reported GWAS loci.

### Enrichment test for GWAS-associated loci.

Fisher’s exact test was applied to test the overrepresentation of quantitative trait loci SNPs in IBD (GWAS catalog, 2020-06-23-EFO_0003767) and cardiovascular disease (GWAS catalog, 2020-06-23-EFO_0000319) SNPs using the height-associated SNPs (GWAS catalog, 2020-08-12-EFO_0004339) as reference (null) set. Specifically, the number of intersections and relative complement of DEGs (in MCI/MC of any trained stimulus vs. RPMI control) and genes within 250 kbp around disease-risk SNPs were tested against the number of intersections and relative complement of those DEGs and genes within 250 kbp around height-associated SNPs with a Fisher’s exact test using R. A *P* value of less than 0.05 was considered significant.

### Quantification and comparison of the cell frequency of clusters or subpopulations.

To describe the abundance and frequency of cell clusters or trained subpopulations, the percentage of cells identified in a cluster or subpopulation out of the total number of cells in each data set were quantified per sample per condition and visualized together in box-and-whisker plots or violin plots. To determine the statistical significance of differences in cell frequency between the different conditions, Dirichlet’s regression model was used (in the R/DirichletReg package) because proportions were not independent of one another.

### Expression variance and distribution.

To identify heterogeneous responses of TI marker genes, we first calculated variances of all expressed genes (defined as log[TP10K + 1] > 0.5) in T2 macrophage clusters. All genes ranked in the top 5% of the observed distribution were regarded as high-variance genes. The variance of TI markers (*TNF*, *IL1B*, *IL6*, *CXCL9*, *CXCL10*, and *CXCL11*) was also compared to the distribution. To minimize the influence of mean expression values on the variance, we drew a distribution of dispersion index (*D* = var/mean) on the same gene sets to validate the results.

### Coexpression analyses.

Spearman’s correlation coefficient for TI markers and other high-variance genes was calculated with R package *Hmisc* for each T2 macrophage subcluster. Genes with 0 counts in a cell were regarded as missing value (NA) in this analysis.

### Assignment of TI subgroups of in vitro monocytes based on marker genes.

In order to further classify trained macrophages, we calculated log(fold change) values of TI markers (*TNF*, *IL1B*, *IL6*, *CXCL9*, *CXCL10*, and *CXCL11*) of each T2 cell from 4 trained stimuli, and then clustered the cells by log(fold change) based on the hierarchical cluster of complete linkage method. Cells were then divided into 3 subgroups based on the branches of the hierarchical clustering tree.

### Cell trajectory analyses and pseudo-time inference.

Monocle 3 (22) is software widely used for scRNA-seq data to order cells according to progression along an unsupervised learning trajectory, and this program also tracks changes as a function of progress along the trajectory, termed pseudo-time inference. To discover the potential development track along the trained cells, we reconstructed single-cell trajectories using Monocle 3. To avoid any possible biological and technical effects in the trajectory construction, we regressed out donor, tissue, stimuli, and MT-gene percentage when aligning the cells in Monocle 3. After the trajectory was constructed, we used the orderCells function to define the unpolarized macrophages as root and starting point of the trajectory, and then the pseudo-time states for the remaining cells were assigned accordingly. Finally, we compared the distribution of pseudo-time states of each trained subgroup, and compared their differences using Wilcoxon’s rank-sum tests.

### Trained subgroup signatures enrichment scores and assignments.

A gene signature enrichment analysis using the AUCell method (24) was applied to link observed gene signatures from 2 trained subgroups to monocytes of existing scRNA-seq studies and in vivo experiments. We ranked selected signature genes with the top 3% of total number of genes in the expression matrix of evaluated studies to calculate a signature score. The maximum possible AUC from the resulting AUC values was normalized to 1 and the values subsequently visualized in violin plots or UMAP plots. To assign subgroups for monocytes from in vivo experiments, thresholds (0.544 for MCI and 0.169 for MC) were estimated from the global distribution of MCI and MC signature scores via the AUCell_exploreThresholds function.

### scRNA-seq profiling of cells prior to restimulation in the in vitro replicate study.

Monocytes were isolated from adherent PBMCs from 3 donors and the in vitro model of TI was performed as described above. To test the incubation effects, cells were stimulated in the training stimulus or with RPMI (control) for 24 hours and rested for 5 days and were sampled prior to the restimulation (pre-T2). To ensure a comparison without batch effects, cells were also sampled 4 hours after the initial RPMI incubation (T1) and 4 hours after LPS restimulation (T2), which served as negative and positive controls, respectively. Subsequently, cells were labelled with Cell Multiplexing Oligo from 10× Genomics following the standard protocol (CG000391 Rev A). Labeled cells were pooled together and loaded into a 10× Genomics Chromium Controller to generate gel beads-in-emulsion (GEMs). Finally, 10× Genomics scRNA-seq libraries were generated according to the manufacturer’s instructions (CG000315 Rev A) and sequenced via NovaSeq 6000 (Illumina).

CellRanger v6.1.1 (10× Genomics) was used to map the reads to the GRCh38 human reference genome and demultiplex the cells from oligo hashtags. In total, 16,683 cells from 13 samples were demultiplexed from 3 pooled libraries (Supplemental Table 1B). The UMI count matrices were then imported to R/Seurat package v3.1 for downstream analyses. For quality control, we excluded genes that were expressed in fewer than 3 cells. We further excluded cells with more than 15 mitochondrial reads, less than 100 or more than 2,000 expressed genes, and less than 500 or more than 7,000 UMI counts. To further filter out the contaminated lymphocytes, we applied SingleR automatic annotation (40) with the main labels from 3 preinstalled reference data sets: Human Primary Cell Atlas data (HPCA), Blueprint Encode data, and Monaco Immune data (Supplemental Figure 8A). Subsequently, the remaining 1,478 monocytes and macrophages were integrated with the initial in vitro data set using the harmony algorithm (41) based on the first 20 principal components to correct technical differences in the gene expression counts of different batches. The cells were then clustered using the Louvain algorithm based on the first 20 harmony dimensions with a resolution of 0.4. Finally, we applied UMAP based on the first 20 harmony dimensions for the integrated visualization, and we applied the above-mentioned AUCell methods for comparing the enrichment scores of trained subgroup signatures at different time points.

### In vivo TI responses (300BCG cohort).

Individuals from the 300BCG cohort were vaccinated in the morning with 0.1 mL of BCG (BCG vaccine strain Bulgaria; Intervax). PBMCs isolated from 3 healthy donors (19, 24, and 25 years of age, all men) before vaccination and 90 days after vaccination were stimulated ex vivo with RPMI medium (control) or 10 ng/mL LPS. In total, 12 samples (3 donors × 2 vaccination-status × 2 restimulation-status) were applied to 10× Genomics scRNA-seq in 1 batch (Supplemental Table 1C).

### Quality control and clustering of in vivo study.

CellRanger v3.1.0 was used to process scRNA-seq of the in vivo study. To generate a digital gene expression matrix for each sample, we mapped their reads to the GRCh38 human reference genome and recorded the number of UMIs. UMI count matrices were then imported to R/Seurat package v3.1 for downstream analyses. For quality control, we excluded genes that were expressed in fewer than 3 cells. We further excluded cells with more than 15 mitochondrial reads, less than 100 or more than 2,500 expressed genes, and more than 10,000 UMI counts. As with the in vitro data, standard LogNormalization in the Seurat package was applied before downstream analysis. PCA was performed based on the 2,000 most variable features identified using the vst method implemented in Seurat. The cells were then clustered using the Louvain algorithm based on the first 20 PCA dimensions with a resolution of 0.4. For 2D data visualization, we performed UMAP also based on the first 20 PCA dimensions.

### Cellular interactions of in vivo PBMCs.

Cell-cell interactions between in vivo PBMC clusters and LPS-restimulated monocytes were estimated using the NicheNet package (29). Ligand-target prior model, ligand-receptor network, and weighted integrated networks were imported from NicheNet data sets (v2) (29). Monocytes were set as receiver/target cell population and all PBMC clusters were set as potential sender/niche. Genes expressed in at least 10% of the cells in one cluster were considered expressed in this cluster. DEGs of in vivo monocytes assigned as trained MCI/MC cells compared with monocytes before BCG vaccination were defined as gene set of interest. Then, potential ligands were ranked based on the presence of their target genes in the gene set of interest. The top-15-ranked ligands and top-predicted-target genes of the top-ranked ligands were inferred and visualized in dot plots and a heatmap.

### Data and materials availability.

Single-cell data have been deposited in the ArrayExpress Archive, which is hosted by the EMBL-EBI, under accession number E-MTAB-9702. Further information about the ArrayExpress can be found on https://www.ebi.ac.uk/arrayexpress/ The code and scripts used in this study are available in GitHub (https://github.com/CiiM-Bioinformatics-group/trained_immunity).

### Statistics.

Statistical analyses were performed with R/Rstudio software (version 3.6.1). *P* values in DE analyses and AUCell score comparisons were calculated using Wilcoxon’s rank-sum test and corrected for multiple testing using Bonferroni’s test. Gene set enrichment analyses (GO and KEGG) were performed with Over Representation Analysis in the R/clusterProfiler package, and corrected for multiple testing using the Benjamini-Hochberg procedure. Dirichlet’s regression model in the R/DirichletReg package was used to determine the statistical significance of differences in cell frequency between the different groups.

### Study approval.

The Human Functional Genomics Project and BCG vaccination studies were approved by the ethical committee of Radboud University Nijmegen (nos. 42561.091.12 and NL58553.091.16). Experiments were conducted according to the principles expressed in the Declaration of Helsinki. Samples of venous blood were drawn after written informed consent was obtained.

## Author contributions

YL and MGN designed the study. SJCFMM, JDA, OB, GK, and ZL performed the experiments and BZ performed data analysis, which was supervised by MGN and YL. JDA, RVC, CJX, and LABJ helped with the data analysis and interpretation of results. BZ, SJCFMM, MGN, and YL interpreted the data and wrote the manuscript with input from all authors. BZ is listed before SJCFMM as co–first author because BZ performed data analysis.

## Supplementary Material

Supplemental data

Supplemental table 1

Supplemental table 2

## Version 1 02/08/2022

In-Press Preview

## Version 2 04/01/2022

Electronic publication
