# Supplementary material for: Single-cell RNA sequencing reveals induction of distinct trained-immunity programs in human monocytes
Source: J Clin Invest. 2022 Apr 1;132(7):e147719. doi: 10.1172/JCI147719 (PMC8970681; doi:10.1172/JCI147719)
Supplement: Supplemental data [file jci-132-147719-s009.pdf]

Figure S1

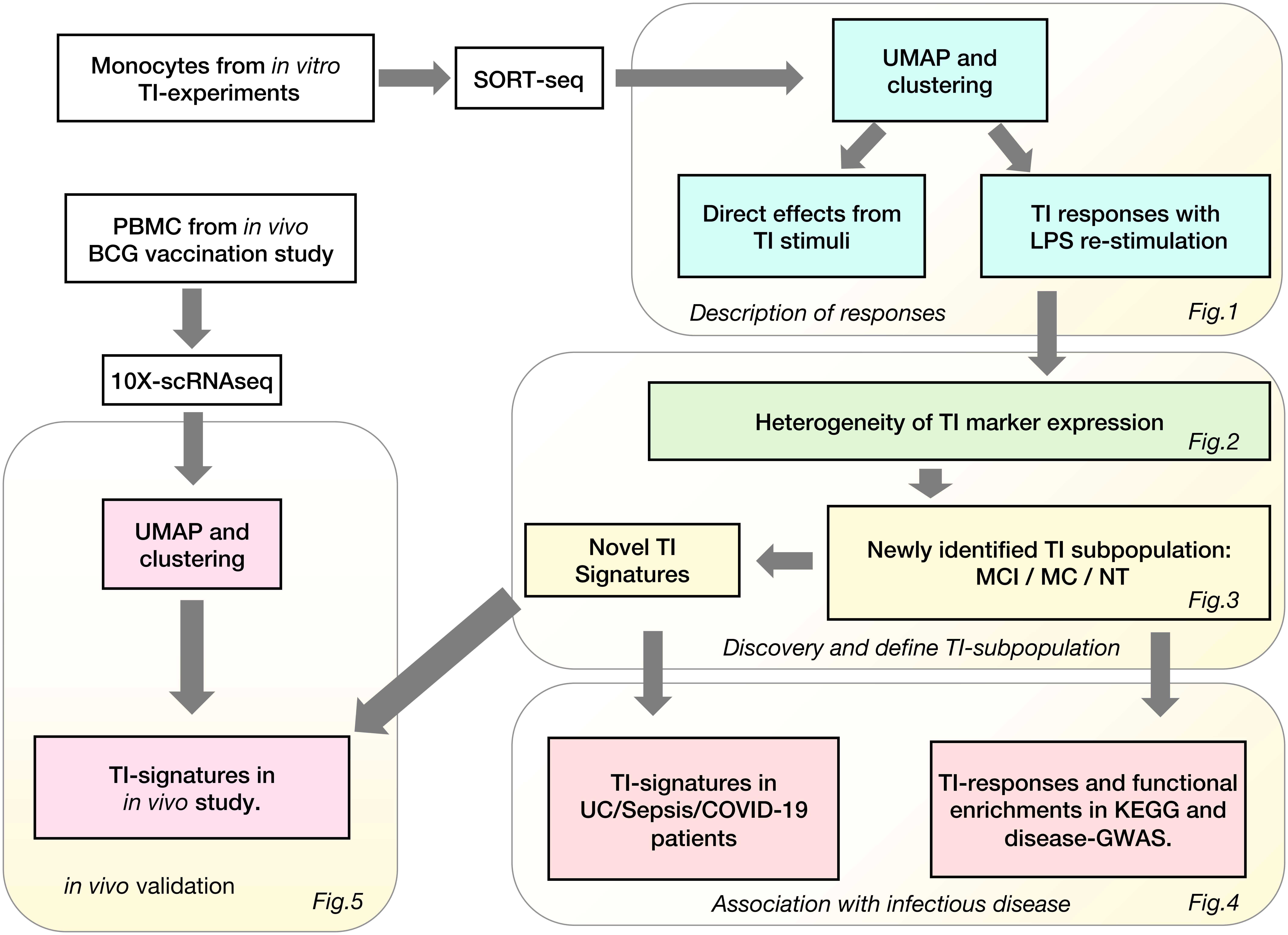

Monocytes from *in vitro* TI-experiments
SORT-seq
UMAP and clustering
Direct effects from TI stimuli
TI responses with LPS re-stimulation
Description of responses
Fig.1
PBMC from *in vivo* BCG vaccination study
10X-scRNAseq
Heterogeneity of TI marker expression
Fig.2
UMAP and clustering
Novel TI Signatures
Newly identified TI subpopulation: MCI / MC / NT
Fig.3
Discovery and define TI-subpopulation
TI-signatures in *in vivo* study.
TI-signatures in UC/Sepsis/COVID-19 patients
TI-responses and functional enrichments in KEGG and disease-GWAS.
in vivo validation
Fig.5
Association with infectious disease
Fig.4

Figure S2

UMAP coloured by 2 time-point, split by three different donors

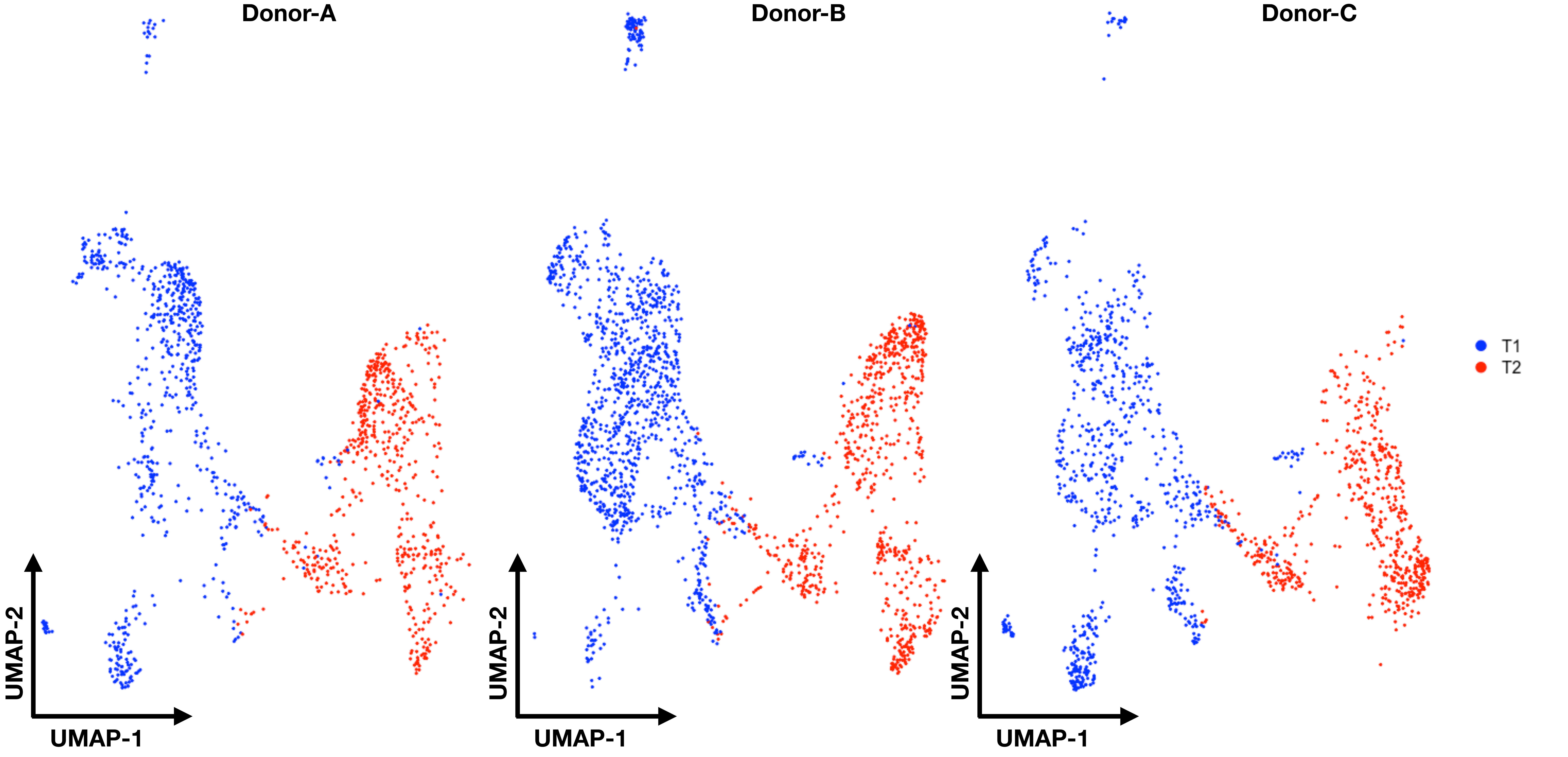

UMAP coloured by sample groups (M-MONO/M-PBMC) and splited by conditions

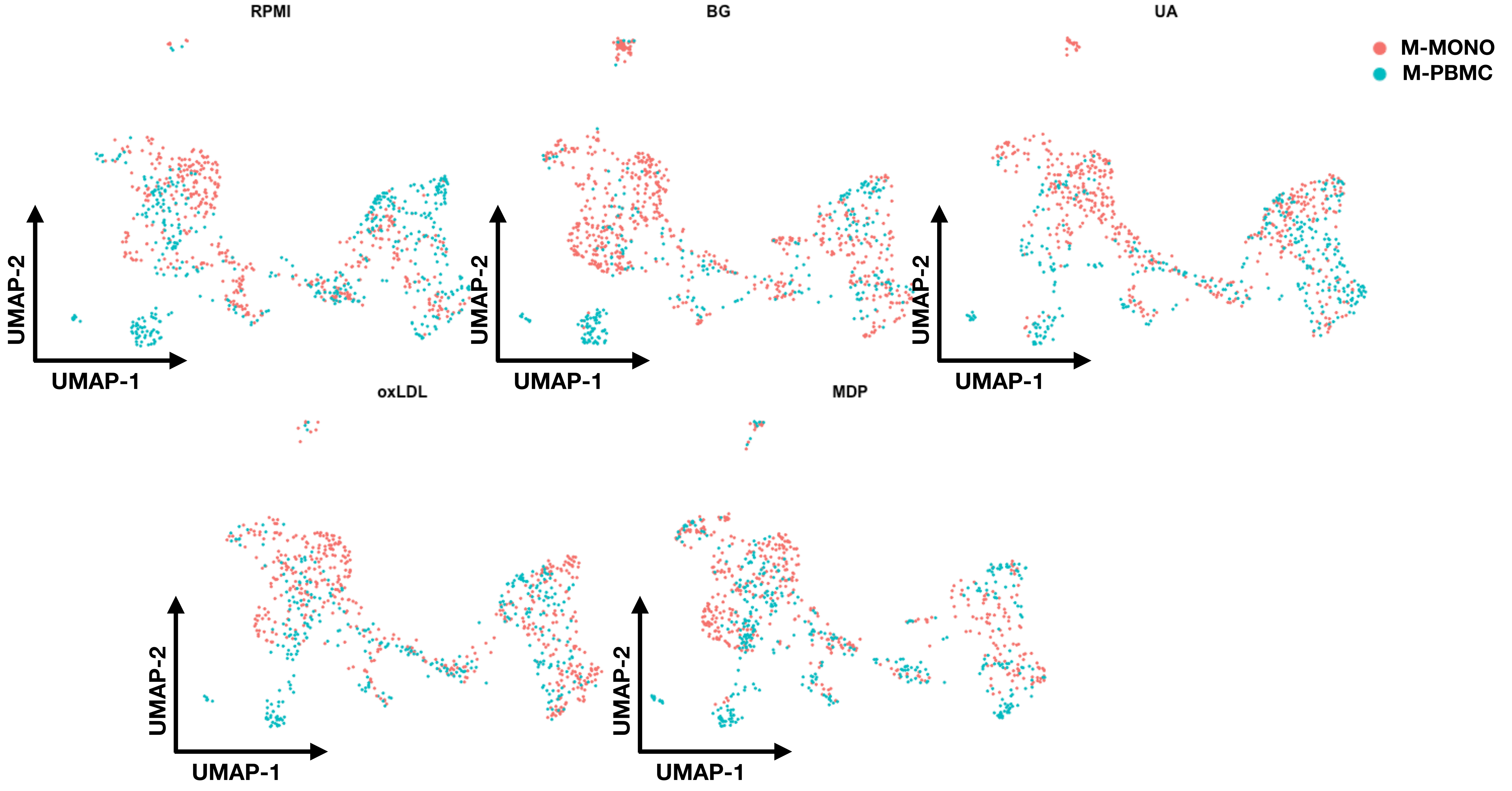

Figure S3

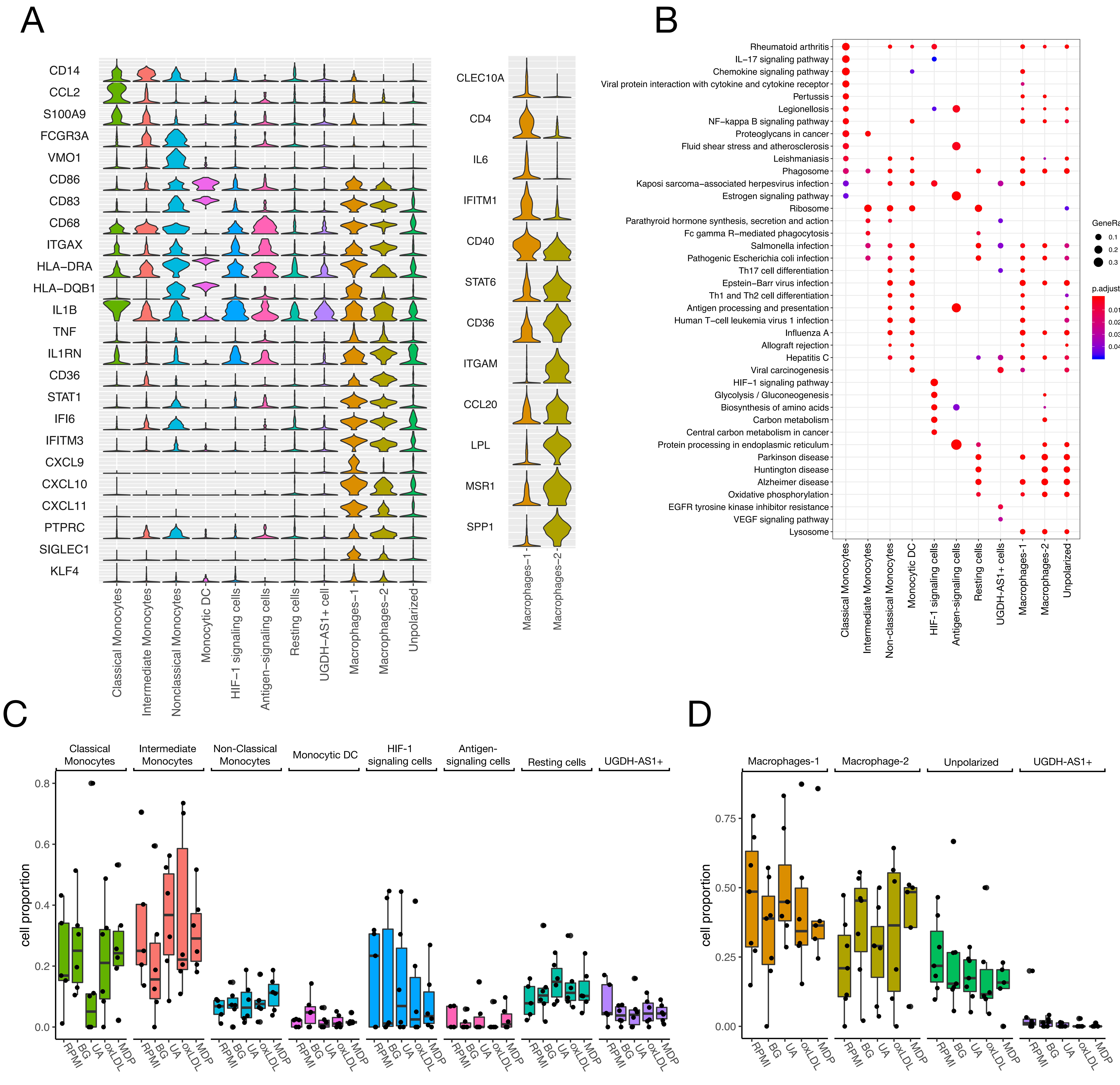
A
CD14
CCL2
S100A9
FCGR3A
VMO1
CD86
CD83
CD68
ITGAX
HLA−DRA
HLA−DQB1
IL1B
TNF
IL1RN
CD36
STAT1
IFI6
IFITM3
CXCL9
CXCL10
CXCL11
PTPRC
SIGLEC1
KLF4
Classical Monocytes
Intermediate Monocytes
Nonclassical Monocytes
Monocytic DC
HIF−1 signaling cells
Antigen−signaling cells
Resting cells
UGDH−AS1+ cell
Macrophages−1
Macrophages−2
Unpolarized
CLEC10A
CD4
IL6
IFITM1
CD40
STAT6
CD36
ITGAM
CCL20
LPL
MSR1
SPP1
Macrophages−1
Macrophages−2
B
Rheumatoid arthritis
IL−17 signaling pathway
Chemokine signaling pathway
Viral protein interaction with cytokine and cytokine receptor
Pertussis
Legionellosis
NF−kappa B signaling pathway
Proteoglycans in cancer
Fluid shear stress and atherosclerosis
Leishmaniasis
Phagosome
Kaposi sarcoma−associated herpesvirus infection
Estrogen signaling pathway
Ribosome
Parathyroid hormone synthesis, secretion and action
Fc gamma R−mediated phagocytosis
Salmonella infection
Pathogenic Escherichia coli infection
Th17 cell differentiation
Epstein−Barr virus infection
Th1 and Th2 cell differentiation
Antigen processing and presentation
Human T−cell leukemia virus 1 infection
Influenza A
Allograft rejection
Hepatitis C
Viral carcinogenesis
HIF−1 signaling pathway
Glycolysis / Gluconeogenesis
Biosynthesis of amino acids
Carbon metabolism
Central carbon metabolism in cancer
Protein processing in endoplasmic reticulum
Parkinson disease
Huntington disease
Alzheimer disease
Oxidative phosphorylation
EGFR tyrosine kinase inhibitor resistance
VEGF signaling pathway
Lysosome
Classical Monocytes
Intermediate Monocytes
Non-classical Monocytes
Monocytic DC
HIF-1 signaling cells
Antigen-signaling cells
Resting cells
UGDH-AS1+ cells
Macrophages-1
Macrophages-2
Unpolarized
GeneRatio
0.1
0.2
0.3
p.adjust
0.01
0.02
0.03
0.04
C
Classical Monocytes
Intermediate Monocytes
Non-Classical Monocytes
Monocytic DC
HIF-1 signaling cells
Antigen-signaling cells
Resting cells
UGDH-AS1+
cell proportion
0.0
0.2
0.4
0.6
0.8
RPMI
BG
UA
oxLDL
MDP
D
Macrophages-1
Macrophage-2
Unpolarized
UGDH-AS1+
cell proportion
0.00
0.25
0.50
0.75
RPMI
BG
UA
oxLDL
MDP

Figure S4

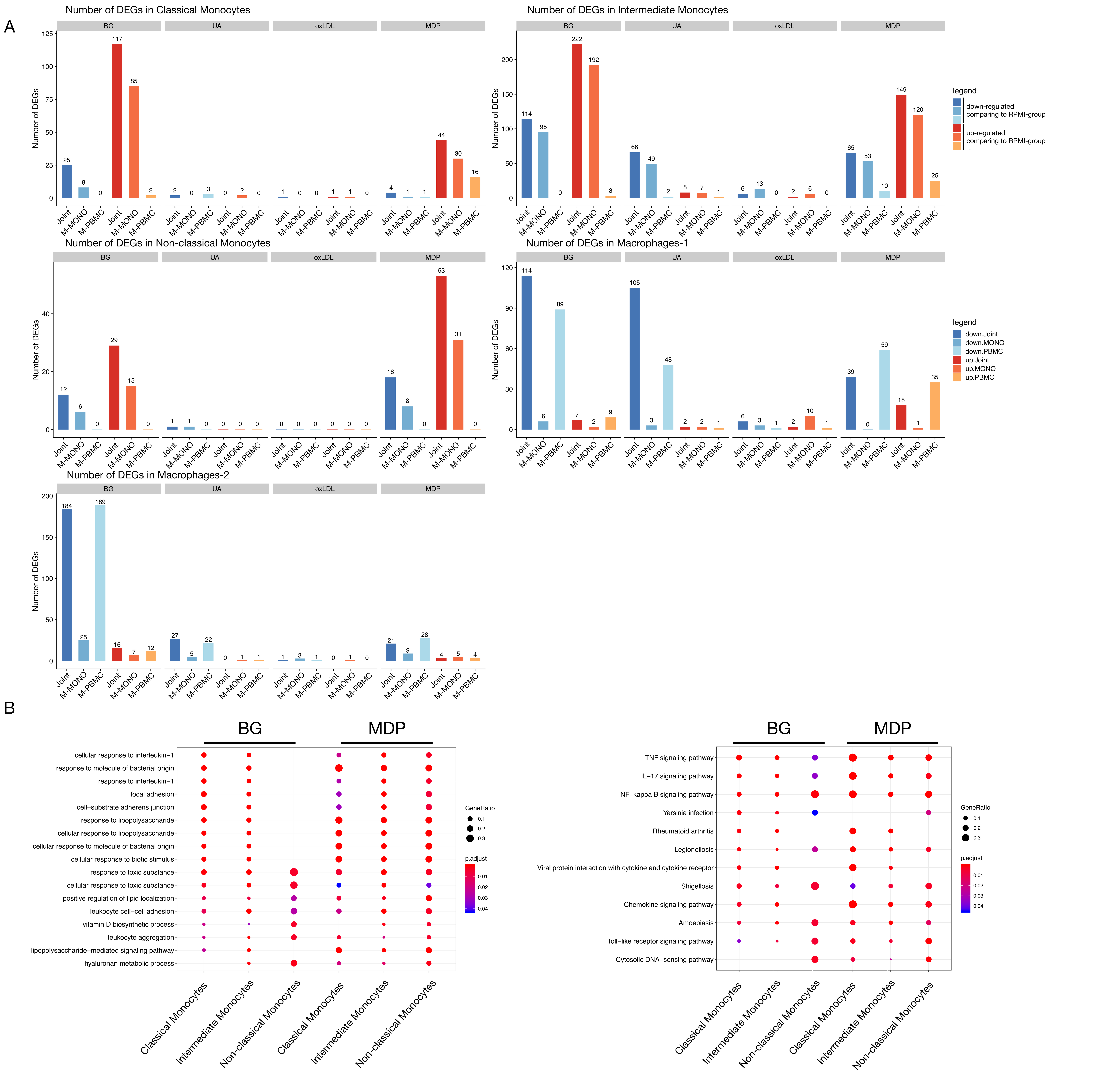

A
Number of DEGs in Classical Monocytes
Number of DEGs in Intermediate Monocytes
Number of DEGs in Non-classical Monocytes
Number of DEGs in Macrophages-1
Number of DEGs in Macrophages-2
BG
UA
oxLDL
MDP
Number of DEGs
Joint
M-MONO
M-PBMC
legend
down-regulated comparing to RPMI-group
up-regulated comparing to RPMI-group
down.Joint
down.MONO
down.PBMC
up.Joint
up.MONO
up.PBMC
B
BG
MDP
cellular response to interleukin−1
response to molecule of bacterial origin
response to interleukin−1
focal adhesion
cell−substrate adherens junction
response to lipopolysaccharide
cellular response to lipopolysaccharide
cellular response to molecule of bacterial origin
cellular response to biotic stimulus
response to toxic substance
cellular response to toxic substance
positive regulation of lipid localization
leukocyte cell−cell adhesion
vitamin D biosynthetic process
leukocyte aggregation
lipopolysaccharide−mediated signaling pathway
hyaluronan metabolic process
GeneRatio
0.1
0.2
0.3
p.adjust
0.01
0.02
0.03
0.04
Classical Monocytes
Intermediate Monocytes
Non-classical Monocytes
TNF signaling pathway
IL−17 signaling pathway
NF−kappa B signaling pathway
Yersinia infection
Rheumatoid arthritis
Legionellosis
Viral protein interaction with cytokine and cytokine receptor
Shigellosis
Chemokine signaling pathway
Amoebiasis
Toll−like receptor signaling pathway
Cytosolic DNA−sensing pathway

Figure S5

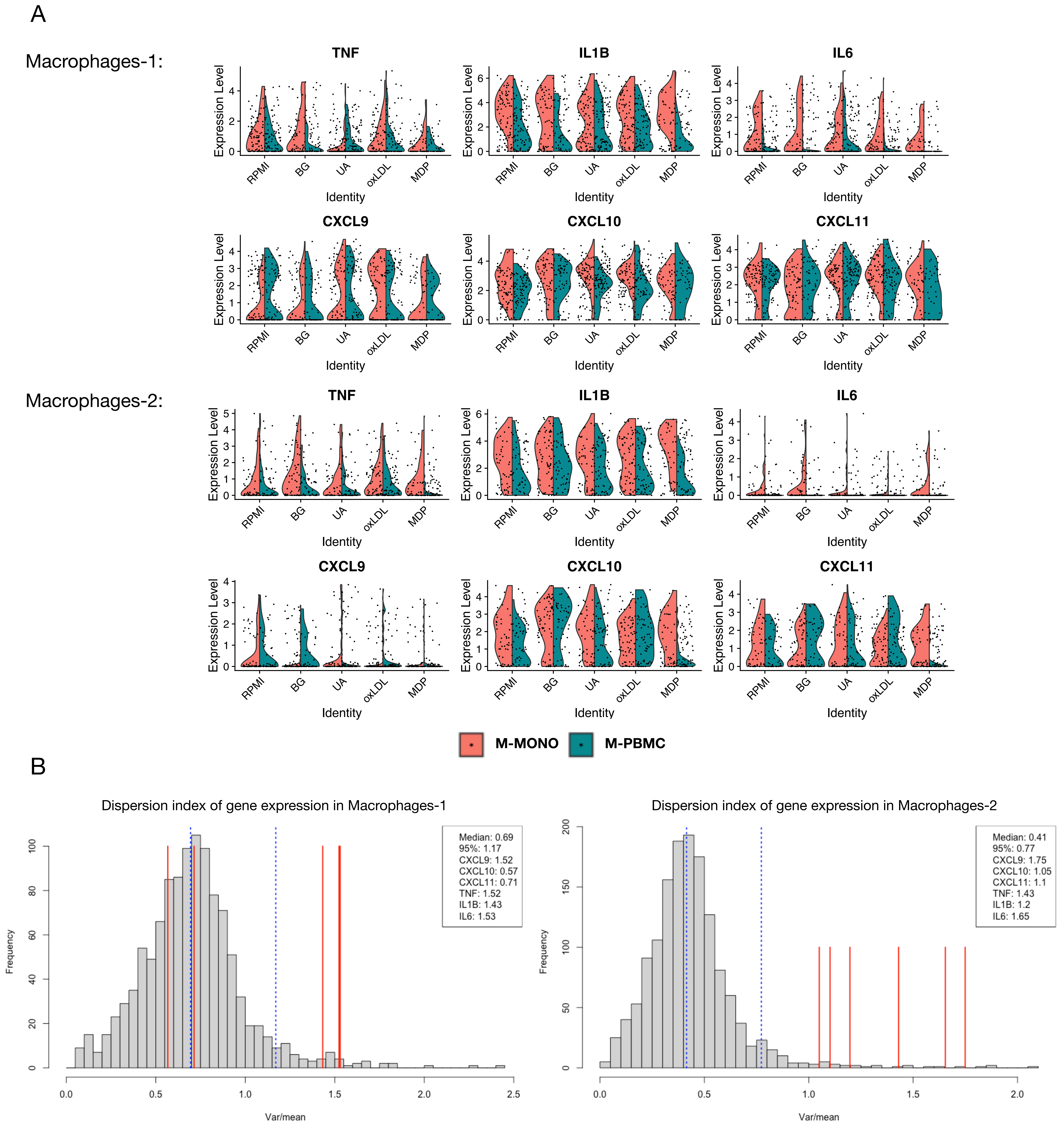
A
Macrophages-1:
TNF
IL1B
IL6
CXCL9
CXCL10
CXCL11
Macrophages-2:
TNF
IL1B
IL6
CXCL9
CXCL10
CXCL11
Expression Level
Identity
RPMI
BG
UA
oxLDL
MDP
M-MONO
M-PBMC
B
Dispersion index of gene expression in Macrophages-1
Median: 0.69
95%: 1.17
CXCL9: 1.52
CXCL10: 0.57
CXCL11: 0.71
TNF: 1.52
IL1B: 1.43
IL6: 1.53
Dispersion index of gene expression in Macrophages-2
Median: 0.41
95%: 0.77
CXCL9: 1.75
CXCL10: 1.05
CXCL11: 1.1
TNF: 1.43
IL1B: 1.2
IL6: 1.65
Frequency
Var/mean

Figure S6

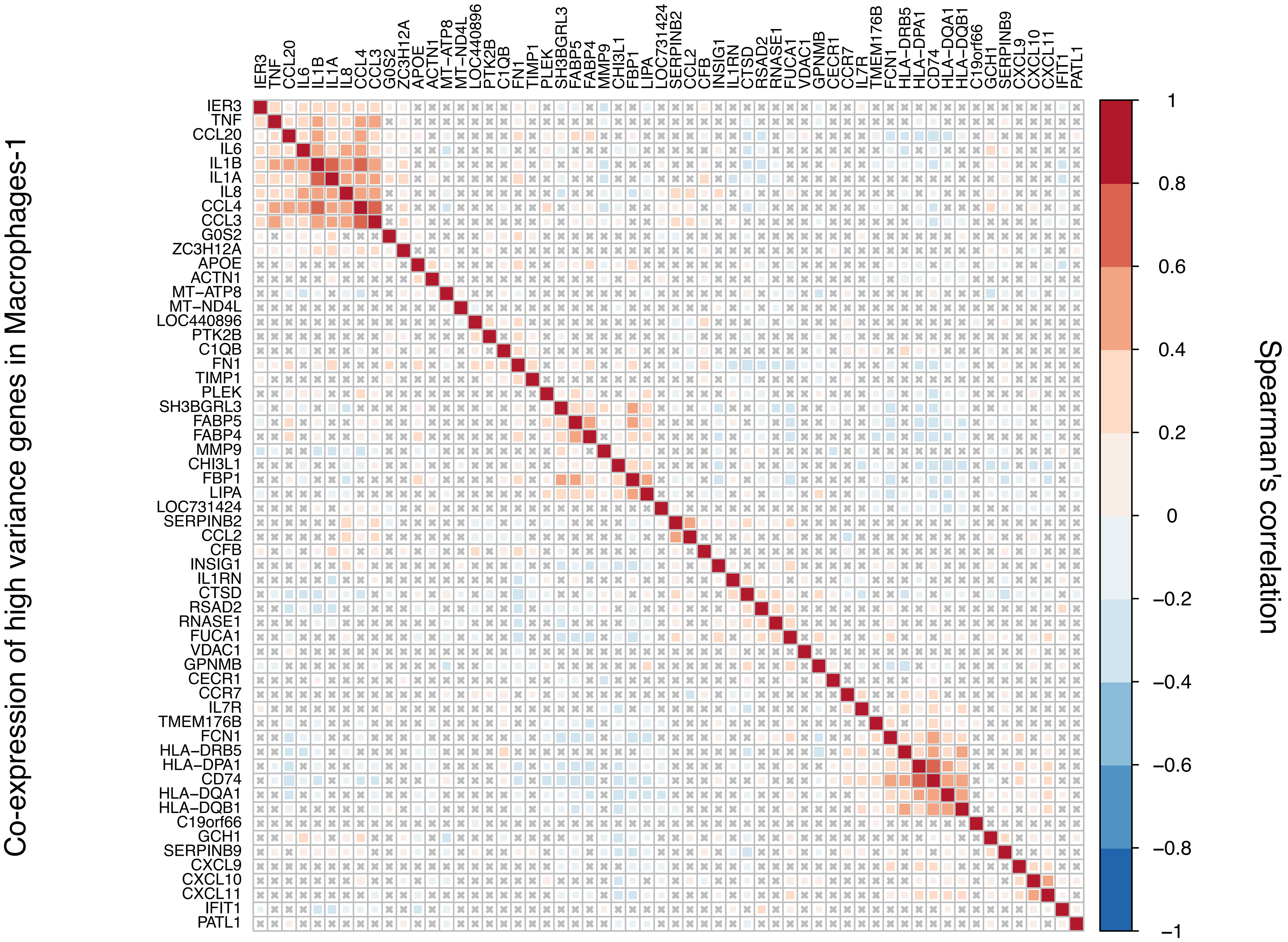
Co-expression of high variance genes in Macrophages-1
IER3
TNF
CCL20
IL6
IL1B
IL1A
IL8
CCL4
CCL3
G0S2
ZC3H12A
APOE
ACTN1
MT−ATP8
MT−ND4L
LOC440896
PTK2B
C1QB
FN1
TIMP1
PLEK
SH3BGRL3
FABP5
FABP4
MMP9
CHI3L1
FBP1
LIPA
LOC731424
SERPINB2
CCL2
CFB
INSIG1
IL1RN
CTSD
RSAD2
RNASE1
FUCA1
VDAC1
GPNMB
CECR1
CCR7
IL7R
TMEM176B
FCN1
HLA−DRB5
HLA−DPA1
CD74
HLA−DQA1
HLA−DQB1
C19orf66
GCH1
SERPINB9
CXCL9
CXCL10
CXCL11
IFIT1
PATL1
Spearman's correlation
1
0.8
0.6
0.4
0.2
0
−0.2
−0.4
−0.6
−0.8
−1

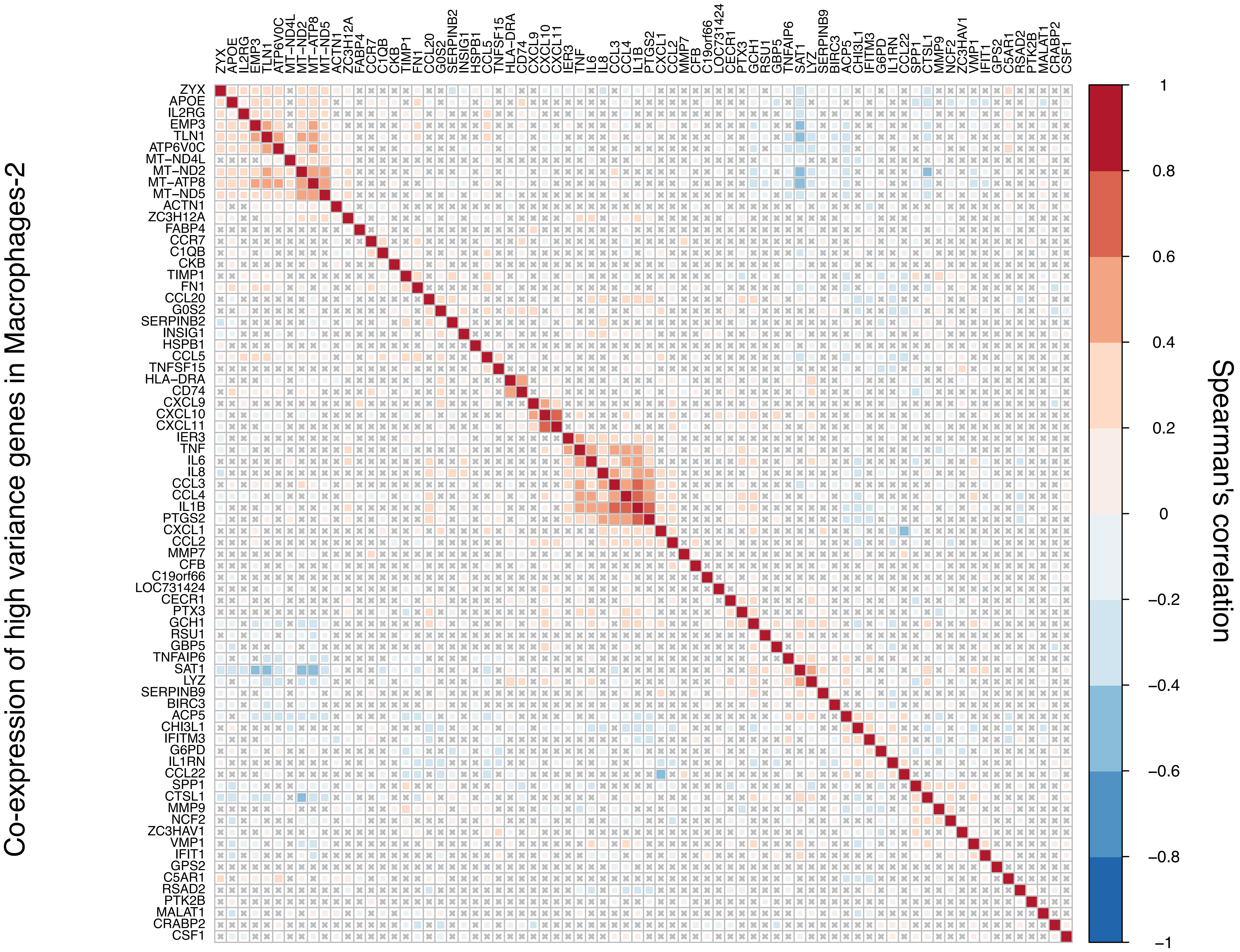
Co-expression of high variance genes in Macrophages-2
ZYX
APOE
IL2RG
EMP3
TLN1
ATP6V0C
MT−ND4L
MT−ND2
MT−ATP8
MT−ND5
ACTN1
ZC3H12A
FABP4
CCR7
C1QB
CKB
TIMP1
FN1
CCL20
G0S2
SERPINB2
INSIG1
HSPB1
CCL5
TNFSF15
HLA−DRA
CD74
CXCL9
CXCL10
CXCL11
IER3
TNF
IL6
IL8
CCL3
CCL4
IL1B
PTGS2
CXCL1
CCL2
MMP7
CFB
C19orf66
LOC731424
CECR1
PTX3
GCH1
RSU1
GBP5
TNFAIP6
SAT1
LYZ
SERPINB9
BIRC3
ACP5
CHI3L1
IFITM3
G6PD
IL1RN
CCL22
SPP1
CTSL1
MMP9
NCF2
ZC3HAV1
VMP1
IFIT1
GPS2
C5AR1
RSAD2
PTK2B
MALAT1
CRABP2
CSF1
Spearman's correlation
1
0.8
0.6
0.4
0.2
0
−0.2
−0.4
−0.6
−0.8
−1

Figure S7

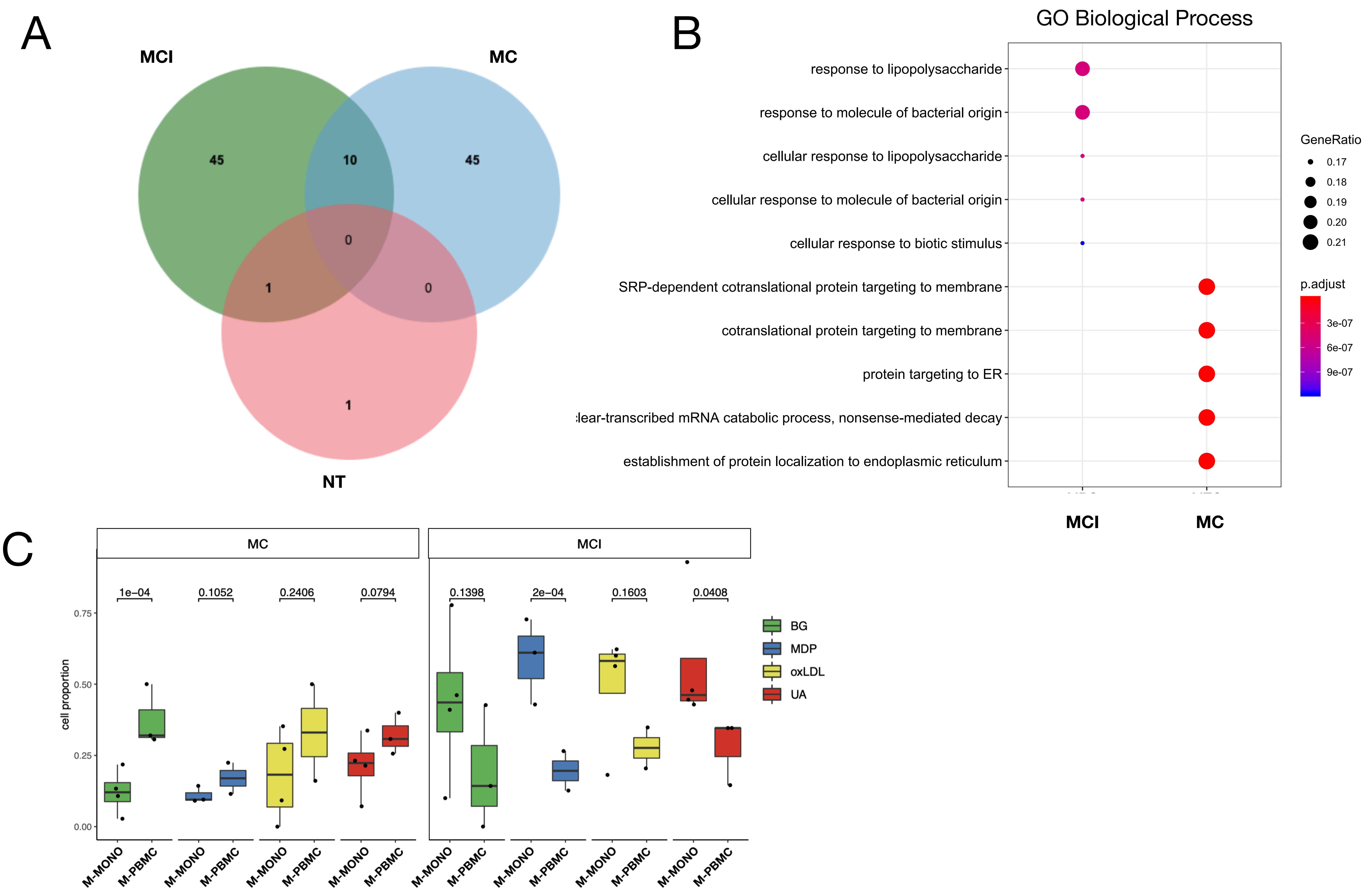
A
MCI
MC
NT
45
10
45
0
1
0
1
B
GO Biological Process
response to lipopolysaccharide
response to molecule of bacterial origin
cellular response to lipopolysaccharide
cellular response to molecule of bacterial origin
cellular response to biotic stimulus
SRP-dependent cotranslational protein targeting to membrane
cotranslational protein targeting to membrane
protein targeting to ER
establishment of protein localization to endoplasmic reticulum
GeneRatio
0.17
0.18
0.19
0.20
0.21
p.adjust
3e-07
6e-07
9e-07
MCI
MC
C
MC
MCI
1e−04
0.1052
0.2406
0.0794
0.1398
2e−04
0.1603
0.0408
cell proportion
0.75
0.50
0.25
0.00
M-MONO
M-PBMC
BG
MDP
oxLDL
UA

Figure S8

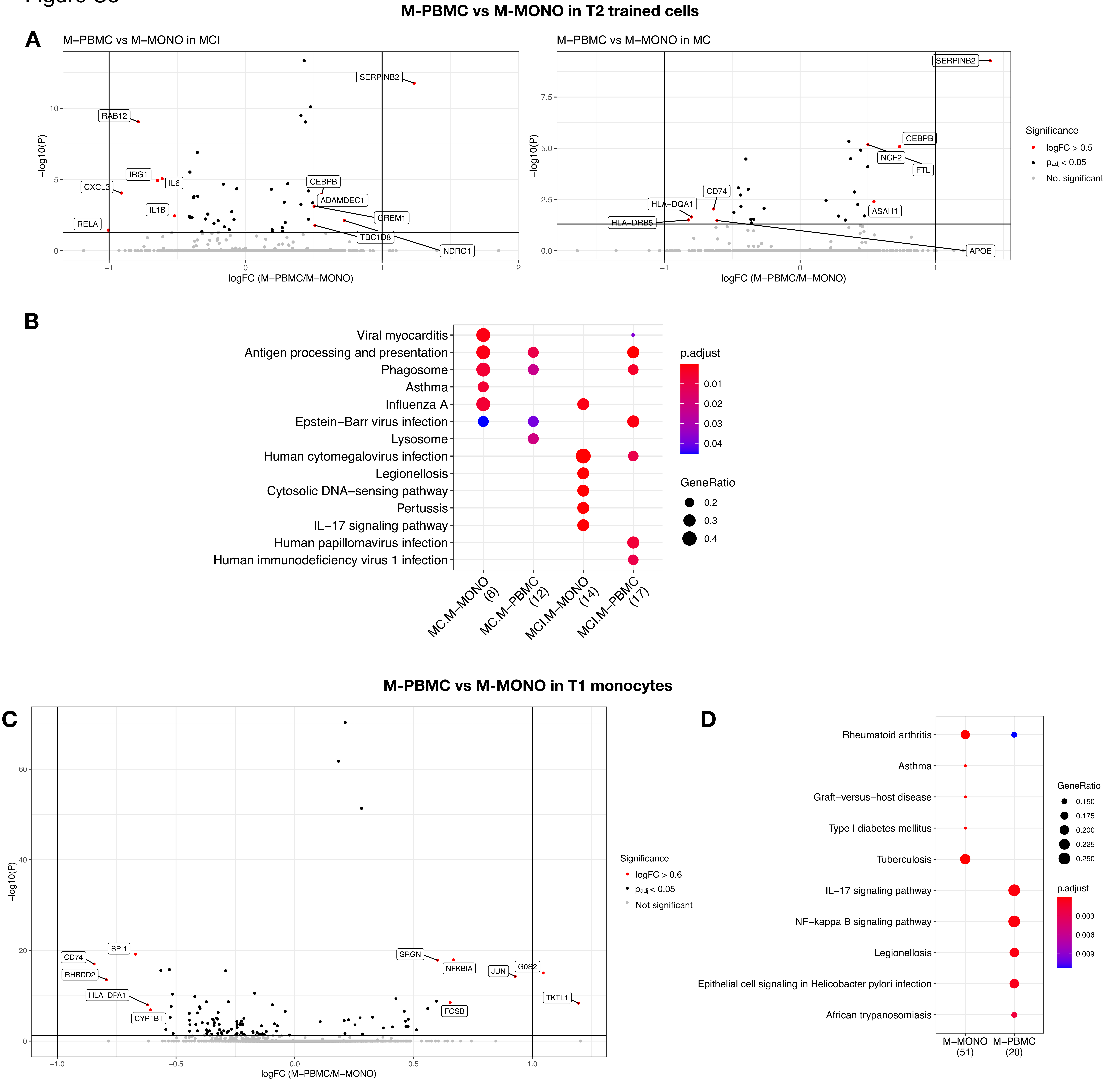
M-PBMC vs M-MONO in T2 trained cells
A
M−PBMC vs M−MONO in MCI
M−PBMC vs M−MONO in MC
−log10(P)
logFC (M−PBMC/M−MONO)
SERPINB2
RAB12
IRG1
IL6
CXCL3
IL1B
RELA
CEBPB
ADAMDEC1
GREM1
TBC1D8
NDRG1
NCF2
FTL
CD74
HLA−DQA1
HLA−DRB5
ASAH1
APOE
Significance
logFC > 0.5
p_{adj} < 0.05
Not significant
B
Viral myocarditis
Antigen processing and presentation
Phagosome
Asthma
Influenza A
Epstein−Barr virus infection
Lysosome
Human cytomegalovirus infection
Legionellosis
Cytosolic DNA−sensing pathway
Pertussis
IL−17 signaling pathway
Human papillomavirus infection
Human immunodeficiency virus 1 infection
MC.M−MONO (8)
MC.M−PBMC (12)
MCI.M−MONO (14)
MCI.M−PBMC (17)
p.adjust
0.01
0.02
0.03
0.04
GeneRatio
0.2
0.3
0.4
M-PBMC vs M-MONO in T1 monocytes
C
−log10(P)
logFC (M−PBMC/M−MONO)
CD74
SPI1
RHBDD2
HLA−DPA1
CYP1B1
SRGN
NFKBIA
JUN
G0S2
FOSB
TKTL1
Significance
logFC > 0.6
p_{adj} < 0.05
Not significant
D
Rheumatoid arthritis
Asthma
Graft−versus−host disease
Type I diabetes mellitus
Tuberculosis
IL−17 signaling pathway
NF−kappa B signaling pathway
Legionellosis
Epithelial cell signaling in Helicobacter pylori infection
African trypanosomiasis
M−MONO (51)
M−PBMC (20)
GeneRatio
0.150
0.175
0.200
0.225
0.250
p.adjust
0.003
0.006
0.009

Figure S9

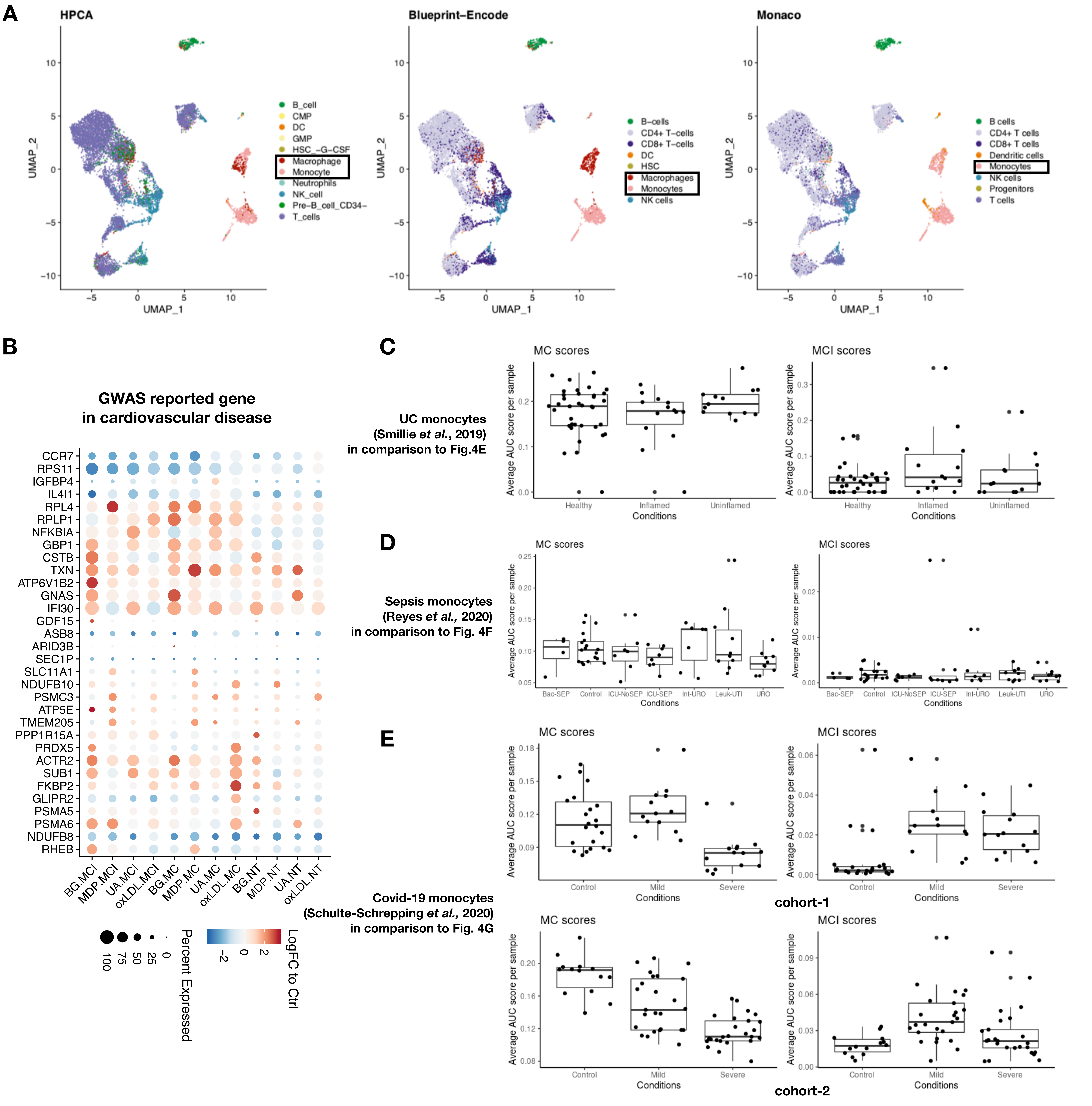

A
HPCA
Blueprint–Encode
Monaco
UMAP_1
UMAP_2
B_cell
CMP
DC
GMP
HSC_-G-CSF
Macrophage
Monocyte
Neutrophils
NK_cell
Pre-B_cell_CD34-
T_cells
B-cells
CD4+ T-cells
CD8+ T-cells
DC
HSC
Macrophages
Monocytes
NK cells
B cells
CD4+ T cells
CD8+ T cells
Dendritic cells
Monocytes
NK cells
Progenitors
T cells
B
GWAS reported gene in cardiovascular disease
CCR7
RPS11
IGFBP4
IL4I1
RPL4
RPLP1
NFKBIA
GBP1
CSTB
TXN
ATP6V1B2
GNAS
IFI30
GDF15
ASB8
ARID3B
SEC1P
SLC11A1
NDUFB10
PSMC3
ATP5E
TMEM205
PPP1R15A
PRDX5
ACTR2
SUB1
FKBP2
GLIPR2
PSMA5
PSMA6
NDUFB8
RHEB
BG.MCI
MDP.MCI
UA.MCI
oxLDL.MCI
BG.MC
MDP.MC
UA.MC
oxLDL.MC
BG.NT
MDP.NT
UA.NT
oxLDL.NT
Percent Expressed
LogFC to Ctrl
C
UC monocytes (Smillie et al., 2019) in comparison to Fig.4E
MC scores
MCI scores
Average AUC score per sample
Healthy
Inflamed
Uninflamed
Conditions
D
Sepsis monocytes (Reyes et al., 2020) in comparison to Fig. 4F
MC scores
MCI scores
Bac-SEP
Control
ICU-NoSEP
ICU-SEP
Int-URO
Leuk-UTI
URO
Conditions
E
Covid-19 monocytes (Schulte-Schrepping et al., 2020) in comparison to Fig. 4G
MC scores
MCI scores
Control
Mild
Severe
Conditions
cohort-1
cohort-2

Figure S10

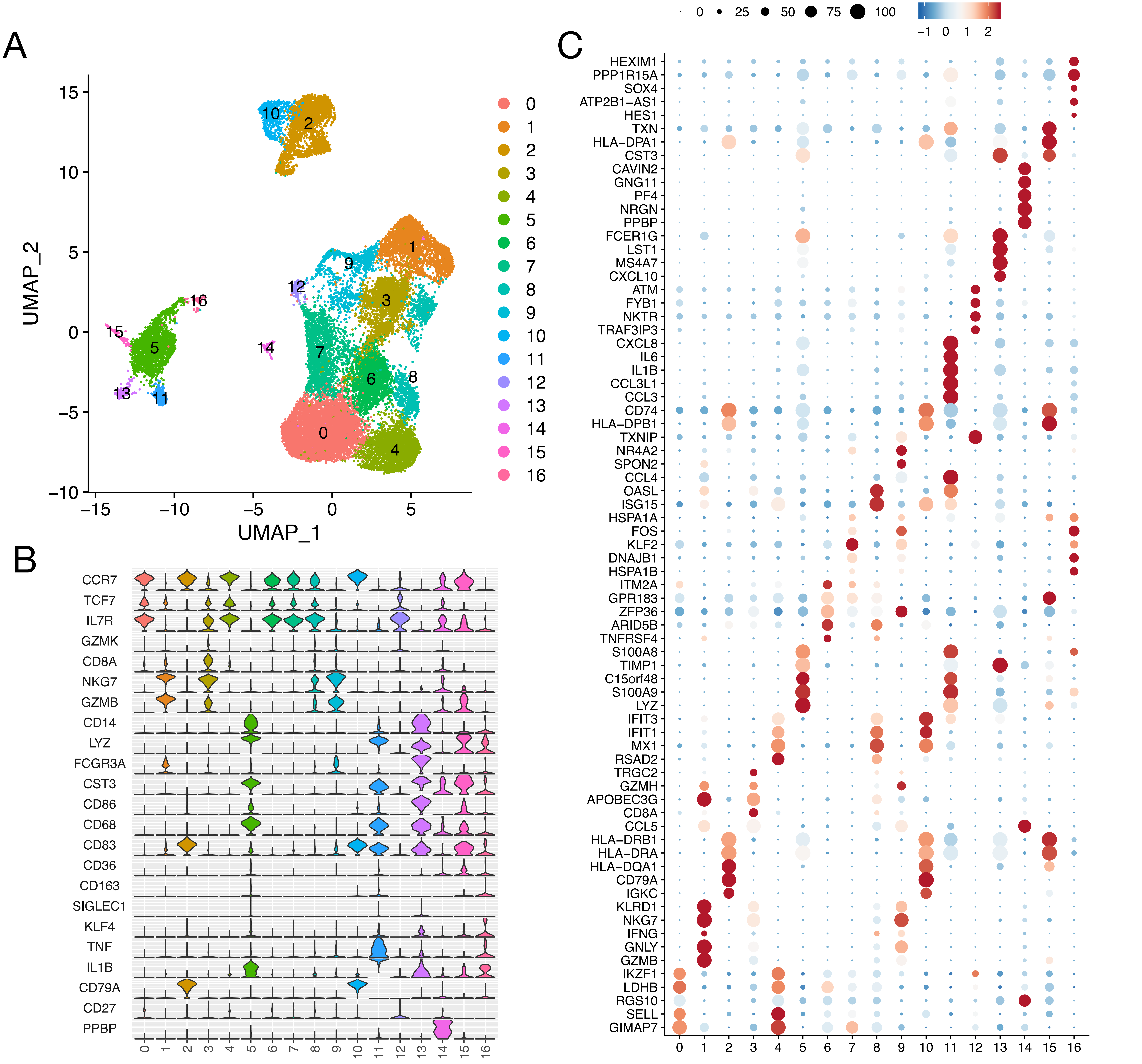
A
B
C
UMAP_1
UMAP_2
0 25 50 75 100
−1 0 1 2
CCR7
TCF7
IL7R
GZMK
CD8A
NKG7
GZMB
CD14
LYZ
FCGR3A
CST3
CD86
CD68
CD83
CD36
CD163
SIGLEC1
KLF4
TNF
IL1B
CD79A
CD27
PPBP
HEXIM1
PPP1R15A
SOX4
ATP2B1−AS1
HES1
TXN
HLA−DPA1
CST3
CAVIN2
GNG11
PF4
NRGN
PPBP
FCER1G
LST1
MS4A7
CXCL10
ATM
FYB1
NKTR
TRAF3IP3
CXCL8
IL6
IL1B
CCL3L1
CCL3
CD74
HLA−DPB1
TXNIP
NR4A2
SPON2
CCL4
OASL
ISG15
HSPA1A
FOS
KLF2
DNAJB1
HSPA1B
ITM2A
GPR183
ZFP36
ARID5B
TNFRSF4
S100A8
TIMP1
C15orf48
S100A9
LYZ
IFIT3
IFIT1
MX1
RSAD2
TRGC2
GZMH
APOBEC3G
CD8A
CCL5
HLA−DRB1
HLA−DRA
HLA−DQA1
CD79A
IGKC
KLRD1
NKG7
IFNG
GNLY
GZMB
IKZF1
LDHB
RGS10
SELL
GIMAP7

Figure S11

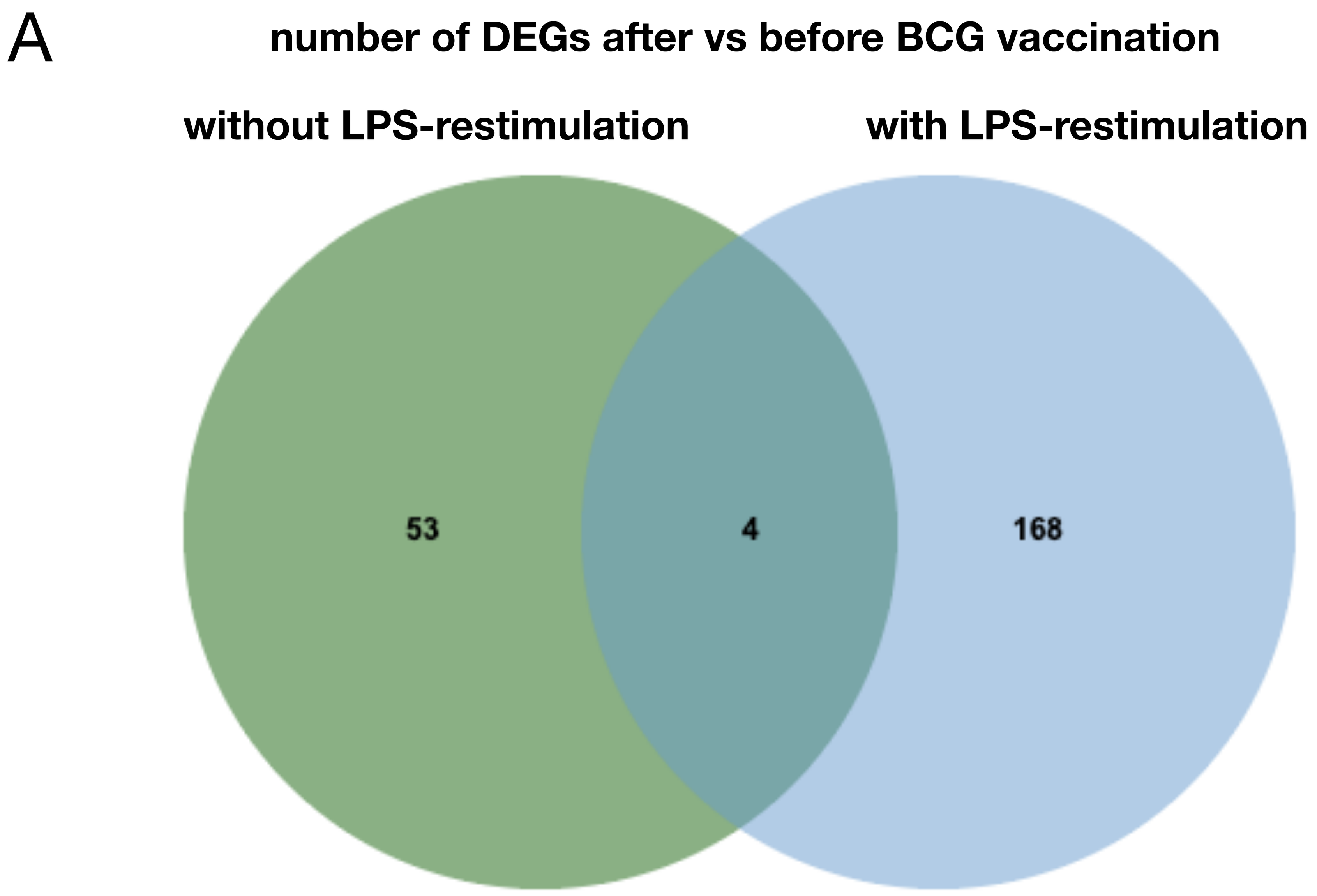
A
number of DEGs after vs before BCG vaccination
without LPS-restimulation
with LPS-restimulation
53
4
168

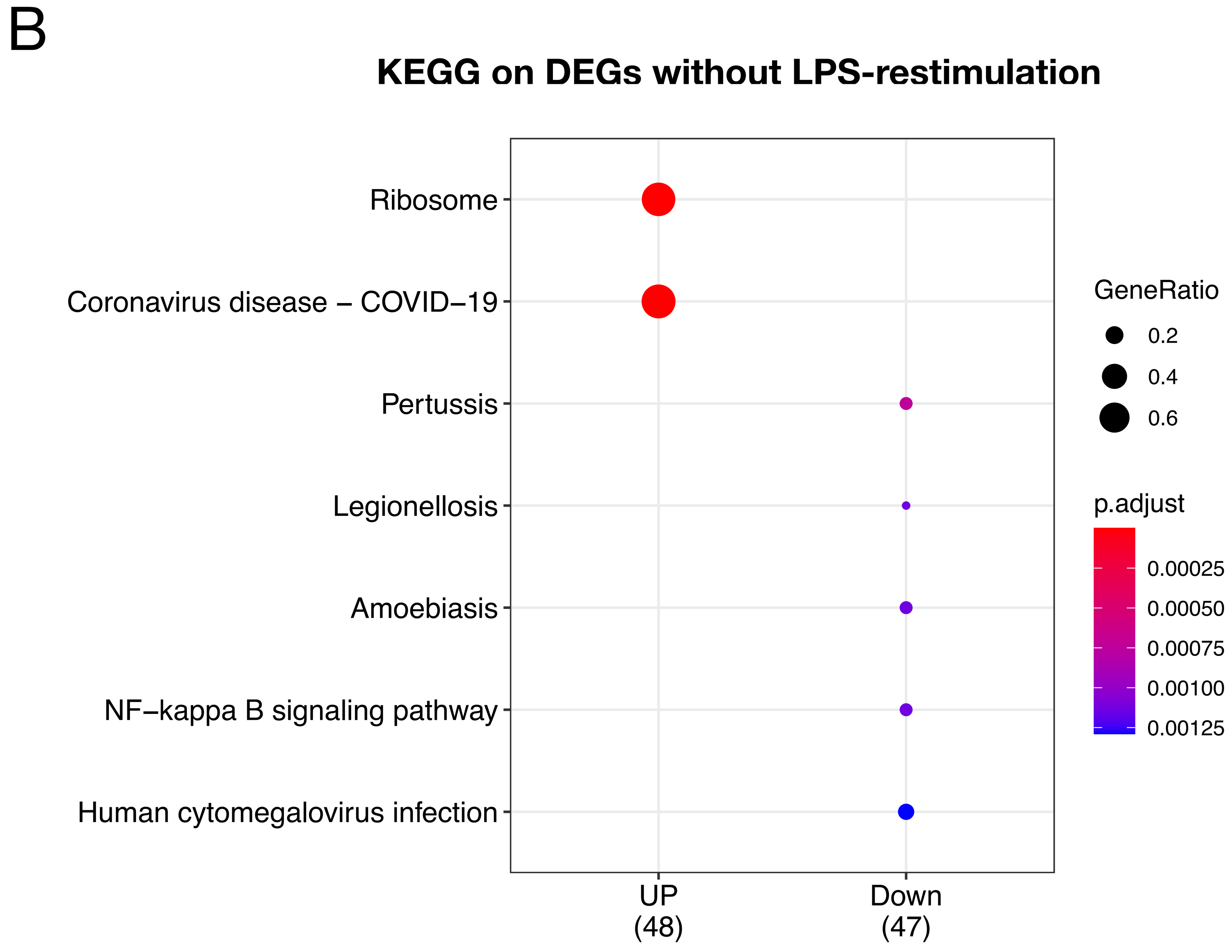
B
KEGG on DEGs without LPS-restimulation
Ribosome
Coronavirus disease – COVID–19
Pertussis
Legionellosis
Amoebiasis
NF–kappa B signaling pathway
Human cytomegalovirus infection
UP (48)
Down (47)
GeneRatio
0.2
0.4
0.6
p.adjust
0.00025
0.00050
0.00075
0.00100
0.00125

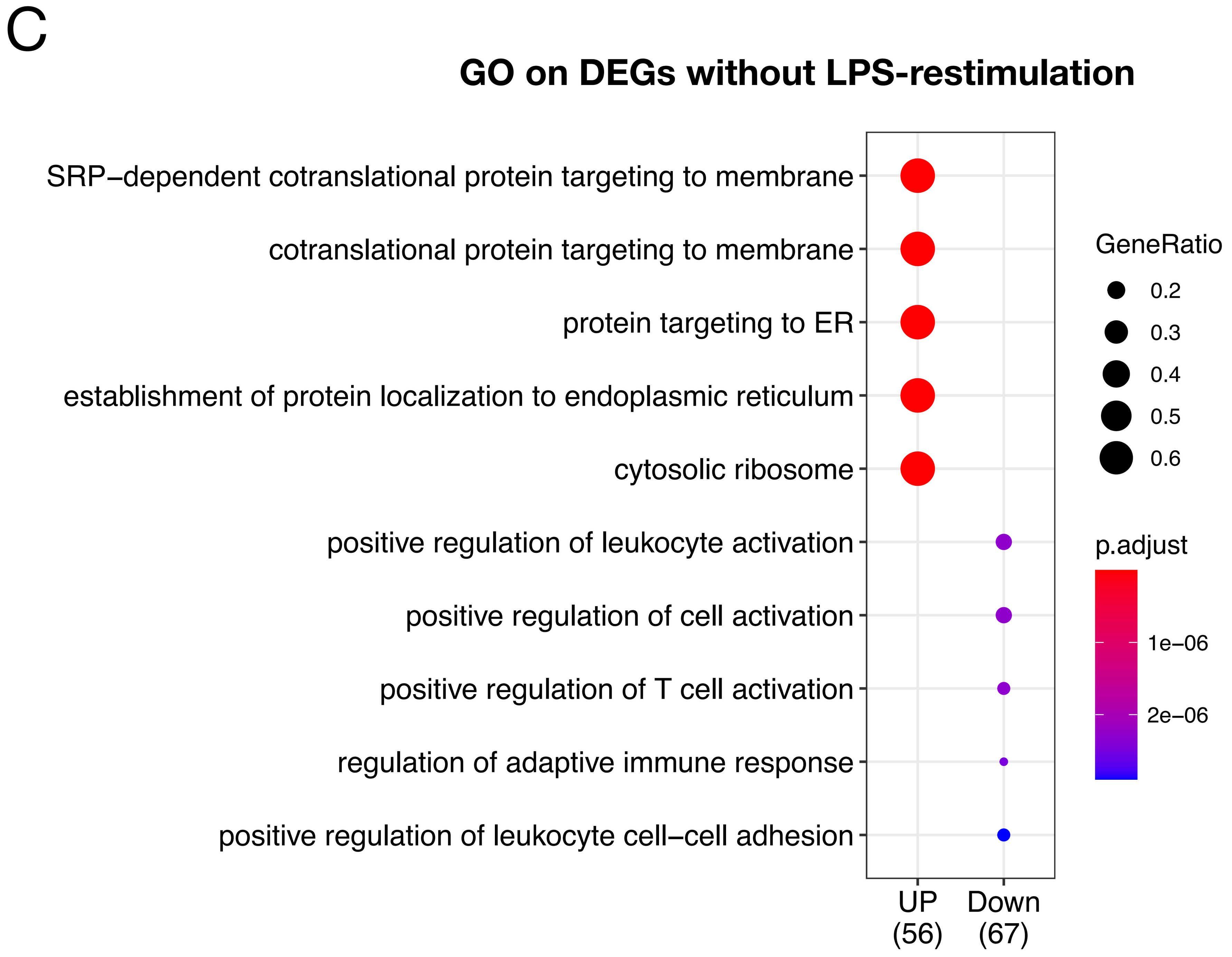
C
GO on DEGs without LPS-restimulation
SRP–dependent cotranslational protein targeting to membrane
cotranslational protein targeting to membrane
protein targeting to ER
establishment of protein localization to endoplasmic reticulum
cytosolic ribosome
positive regulation of leukocyte activation
positive regulation of cell activation
positive regulation of T cell activation
regulation of adaptive immune response
positive regulation of leukocyte cell–cell adhesion
UP (56)
Down (67)
GeneRatio
0.2
0.3
0.4
0.5
0.6
p.adjust
1e–06
2e–06
